# Effects of Legume‒Cereal Rotation on Sorghum Rhizosphere Microbial Community Structure and Nitrogen‐Cycling Functions

**DOI:** 10.1002/mbo3.70085

**Published:** 2025-10-21

**Authors:** Ben Jesuorsemwen Enagbonma, David Mxolisi Modise, Olubukola Oluranti Babalola

**Affiliations:** ^1^ Food Security and Safety Focus Area, Faculty of Natural and Agricultural Sciences North‒West University Mmabatho South Africa; ^2^ Faculty of Natural and Agricultural Sciences North‒West University Potchefstroom South Africa; ^3^ Department of Life Sciences, Imperial College London Silwood Park Campus Ascot Berkshire UK

**Keywords:** crop yield, legume rotation, shotgun metagenomics, sustainable agriculture, synthetic inputs

## Abstract

Legumes form mutualistic interactions with specific soil microbiomes that fix atmospheric nitrogen and improve soil fertility. However, legume‐based rotations influence on soil microorganisms and their correlations with soil physicochemical parameters during subsequent crop development are not yet clear. We examined the shifts in microbial community structure and nitrogen genes via shotgun sequencing across cowpea–sorghum, soybean–sorghum, maize–sorghum rotations, and sorghum without precrops. Precropping in rotation significantly affected N‐NO_3_, clay, and silt, and caused a shift in the rhizosphere microbiome. Actinomycetota was the most predominant bacteria across all the cropping systems, followed by Pseudomonadota, whose composition differed across the cropping systems. Legume in rotation increased the relative abundance of *Streptomyces* and reduced the relative abundances of *Pyxidicoccus*, *Microbacterium*, and *Microvirga. Nocardioides* and *Solirubrobacter* predominated in the soil after the maize crops. Shannon index, non‐metric multidimensional scaling, and permutational multivariate analysis of variance revealed that crop rotation caused significant differences in both the alpha and beta diversity of the microbial community and the nitrogen‐cycling functional genes. The relative abundances of *amoC*, *narH*, *gltB*, *glnA*, *ureC*, *napA*, and *napA* significantly increased in legume monocrops in rotation. The relative abundances of *glnA*, *gltB*, *narZ*, and *narH* increased in the soil after maize cropping, whereas sorghum without precrops significantly increased the relative abundances of *glnA*, *narZ*, and *ureC*. Several soil physicochemical parameters drive microbial communities. *S, Na, N‐NH_4_, N‐NH_3_, and P were the most significant environmental variables regulating microbiome and nitrogen‐cycling genes by crop rotation. This study supports sustainable agricultural practices and promotes sorghum development through rhizosphere microbiome optimization.

## Graphical abstract

Our metagenomic study revealed the impact of sorghum cultivated in soils previously grown with cowpea, soybean, and maize on rhizosphere microbiome and soil nitrogen cycling.

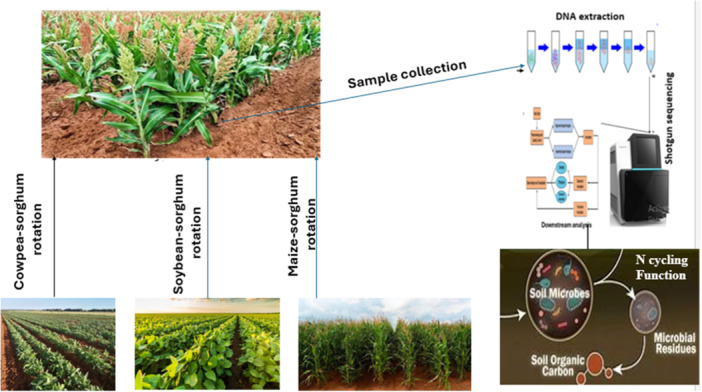

## 1 Introduction

One key goal of farming is to increase the quality and quantity of crop production and conserve soil fertility (Amoo et al. 2021). Diversifying crops and integrating them into soil are among the vital schemes that aid the accomplishment of these agendas, which concurrently lessen the effects of farming on climate and negative ecological shifts (Babalola et al. 2025). It has been established that legumes, which are crops with typical nitrogen‐fixing abilities, play a crucial role in crop rotation (Babalola and Enagbonma 2025a). The significant advantages of crop rotation include improvements in soil physical, chemical, and biological parameters as well as soil health (Uzoh et al. 2019). It also improves weed control, curbs pests and pathogens, and increases nutrient availability, soil fertility, harvest, and output (Shah et al. 2021). The use of legumes as cover crops or their inclusion in mixed cropping or crop rotation impacts the physical, chemical, and biological parameters of soils (Mupangwa et al. 2021). With respect to plant nutrition, the nitrogen level of legumes is affected by their symbiotic nitrogen fixation and by the mobilization of phosphorus (Zhong et al. 2023). The influences of legumes on the enhancement of diverse succeeding crops, such as rice (S. Wang et al. 2022), barley (Pappa et al. 2012), maize (Ojiem et al. 2014), and wheat (Geng et al. 2023), in terms of quality and yield are well reported. Furthermore, the positive impact of legumes resulting from symbiosis with arbuscular mycorrhizal fungi (X.‐Q. Liu et al. 2023) enhances plant water and nutrient uptake. A study by N'Dayegamiye et al. (2015) reported that four legume‐based systems (crimson clover, hairy vetch, alfalfa, and hairy vetch/wheat) markedly enhanced soil stability and dehydrogenase and alkaline phosphatase enzyme activities. It has also been reported that legume systems significantly increase the yield and nitrogen uptake of corn and wheat.

Crop rotation with legume integration can reduce the effect of farming on climate variability by reducing the use of chemical fertilizers needed for subsequent crops through the addition of nitrogen from biological fixators into the farming system (Hassen et al. 2017). In this way, a decrease in energy demand in nitrogen fertilizer production is achieved (Zhao et al. 2024). Additionally, a decrease in nitrous oxide discharge in cropping systems caused by legumes was demonstrated. Compared with monocropping, crop rotation with legume crops intensifies carbon sequestration in the soil (Matthews et al. 2025).

Among all cereal precrops, legumes such as soybean (*Glycine max* (L.) Merr.) and cowpea (*Vigna unguiculata* (L.) Walp.) are the best (Allie et al. 2025), and as such, agriculturalists are guided by their application when choosing specific cereal species in rotation (Ghosh et al. 2020). Cereal–cereal rotations have a profound effect on ecological function, and sustainable farming must depend on alternative approaches, like, legume‐based rotation (Tzemi et al. 2024). Thus, in fields, cereals such as sorghum should typically be planted after suitable precrops. Sorghum (*Sorghum bicolor*) is the fifth most common bioenergy crop (Bakari et al. 2023); it was originally domesticated in sub‐Saharan Africa but is now cultivated worldwide (Ananda et al. 2020). Its large distribution is tied to its ability to withstand poor soil conditions as well as its resilience to drought, especially in arid and semiarid regions (Khalifa and Eltahir 2023), hence it was used as a model, in this study. Sorghum is a major food in Africa because of its nutritional benefits and often contributes more effectively than maize (*Zea mays* L.) to household food security (Pambuka et al. 2021). The demand for sorghum in recent years has increased because of climate change and the ever‐increasing human population (Tonapi et al. 2020). To meet this high demand for sorghum plants, farmers have relied on agrochemicals that provide short‐term benefits. However, the prolonged and excessive use of chemical fertilizers and pesticides to increase sorghum production (Akhtar et al. 2020; Amoo et al. 2021) could lead to soil nutrient decline, soil erosion, water and air pollution, and a decline in biodiversity (Babalola and Enagbonma 2024; Enagbonma et al. 2024). Owing to the environmental problems linked to synthetic inputs, research has called for the use of ecofriendly strategies in farming (Enagbonma et al. 2024; Enagbonma and Babalola 2019).

In agricultural ecosystems, one way to increase soil organic matter and soil nutrients is by returning crop residues to the soil (L. Liu et al. 2024). Plant residues are loaded with magnesium, calcium, potassium, phosphorus, and nitrogen and are a renewable resource of nutrients (Torma et al. 2018). This integration of plant residues could increase nutrient status, increase porosity, enhance soil structure, decrease bulk density, and similarly increase the biodiversity of soil microorganisms (Fu et al. 2021). The soil microbiome influences disease control and suppression, carbon sequestration, nutrient cycling, organic matter transformation, plant residue decomposition, and soil structure maintenance and therefore controls soil productivity and fertility (Fu et al. 2021; Jiao et al. 2018). Microbial abundance and biological activity are altered by the integration of crop residues into the soil, and the fates of these alterations are closely linked to residue dose, residue composition, and plant species (P. Wang, Xie, et al. 2023; Y. Wang, Zhang, et al. 2023). Compared with fallow sorghum, legume residue has a significant effect on the growth and yield of sorghum (Shuaibu et al. 2015) and has greater fungal and bacterial biomass in the sorghum rhizosphere (Marschner et al. 2004; Jalloh et al. 2024). The rhizosphere microbiome contributes significantly to basic nitrogen‐cycling processes such as ammonium production via dissimilatory nitrate reduction, anammox, denitrification, mineralization, nitrification, and nitrogen fixation (Fan et al. 2024). These nitrogen‐cycling processes are driven by specific gene families (*nrfA*, *hzsB*, *nosZ*, *nirS*, *nirK*, *napA*, *narG*, *amoA*, and *nifH*) that catalyze the chemical reactions involved (Yan et al. 2024).

Given the intricate nature of nitrogen cycling in soils, investigating only one or a few nitrogen genes is inadequate to capture the entire variation in the nitrogen‐cycling gene network within agricultural ecosystems (Shu et al. 2024). This constraint prevents us from gaining a full understanding of how cropping systems impact the dynamics of nitrogen cycles in soils. Therefore, the focus of this study is to examine how cultivating sorghum in soils that have been previously planted with legumes impacts the functionality of the rhizosphere microbiome and soil nitrogen cycling. Specifically, this study aims to (i) assess the influence of different precrop types (cowpea, soybean, and maize) on soil physicochemical parameters, (ii) investigate the impact of different precrop types (cowpea, soybean, and maize) on the soil microbial community, (iii) explore the impact of different precrop types (cowpea, soybean, and maize) on the soil microbial functional genes involved in nitrogen cycling, and (iv) evaluate the relationships between the physicochemical properties of the soil and the microbial community and nitrogen‐cycling genes. Since several researchers have reviewed the ability of legumes to enhance soil health through nitrogen fixation and the addition of organic matter, this study postulated that (a) cultivation in soils preceded by legume systems results in greater functional diversity (Functional diversity generally involves understanding communities and ecosystems based on what organisms do, rather than on their evolutionary history, Petchey and Gaston 2006) of soil microbial communities, leading to enhanced impacts on nitrogen cycling, and (b) the increased diversity of belowground microbiome functionality is driven by nitrogen‐related forms (N‐NO_3_ and N‐NH_4_). To investigate these premises, we carried out a year field experiment on North‒West University's farm and compared sorghum cultivation in soils preceded by the cultivation of two legumes (cowpea and soybean) with sorghum cultivation in soils without previous legume cultivation. In this study, the idea of comparing soybean–sorghum rotation, cowpea–sorghum, and maize–sorghum rotation is based on Hamza and Akinrinde's (2016) research, which shows that legume‐sorghum rotation system focuses on soil fertility, nitrogen enrichment, and organic matter, which lead to stronger yield improvements. While maize–sorghum rotation focuses on soil structure and moisture conservation, but with limited direct nutrient carryover. This study offers insight into the benefits of crop diversification, specifically legume integration, on microbiome functionality and biological activity in soil.

## 2 Materials and Methods

### 2.1 Sampling Location and Experimental Field

The field research took place at North‒West University's farm, which is located in Molelwane in the North‒West Province of South Africa (25°49′22″ S and 25°36′54″ E) at an altitude of 1286 m. The temperature varies between 27°C and 38°C in summer and between 11°C and 18°C in winter. The area receives an annual rainfall of 200–450 mm, most of which falls during the summer. The experimental plots had soil characterized by sand (80%), clay (17%), and silt (2%) contents in the 0–20‐cm soil layer. The moisture content of the soil in the experimental plots ranged from 3.28% to 7.64%, and the pH ranged from 5.58 to 6.36. Additional information on the soil properties of the experimental field conditions can be found in Table 1. In 2023, an experimental field of approximately 2000 m^2^ was divided into eight plots. The first and second plots, designated G1 and G2, had previously been used for cowpea (PAN311: cultivar) monocropping for 5 months. The third and fourth plots, designated G5 and G6, had previously been used for soybean (Y657: cultivar) monocropping for 5 months. The fifth and sixth plots, designated G7 and G8, had previously been used for maize (CRN‐3505: cultivar) monocropping for 4 months. At maturity, the cowpea, soybean, and maize crops were harvested thereafter, and their plant residues (stems and stripped pods of the legumes and straw of maize) were collected, sliced (4 cm long pieces), and integrated into the soil to a depth of 20 cm in November 2023. The seventh and eighth plots, designated G4 and G4, had no precrop or plant residues. We waited till February before commencing the experimental treatments because fresh residues need time to decompose and mineralize so that nutrients become available to the next crop (Niewiadomska 2025).

**Table 1:** Effects of crop rotation on soil physicochemical parameters.

Parameter (unit)	Soil sample							
	G1	G2	G3	G4	G5	G6	G7	G8
pH (KCl) 01:02.5	5.80 ± 0.36^a^	6.00 ± 0.45^a^	6.00 ± 0.37^a^	6.01 ± 0.33^a^	6.36 ± 0.29^a^	5.90 ± 0.57^a^	6.07 ± 0.27^a^	5.58 ± 0.53^a^
P Bray 1 (mg/kg)	11.00 ± 2.65^a^	17.67 ± 12.50^a^	13.33 ± 2.08^a^	11.00 ± 4.36^a^	12.33 ± 2.08^a^	12.33 ± 2.08^a^	10.67 ± 2.08^a^	11.00 ± 1.73^a^
*S value	4.26 ± 0.61^a^	4.31 ± 0.70^a^	4.14 ± 0.58^a^	4.11 ± 0.46^a^	4.60 ± 0.25^a^	4.05 ± 0.70^a^	4.19 ± 0.38^a^	3.81 ± 0.38^a^
K (mg/kg)	13.43 ± 4.31^a^	13.27 ± 4.59^a^	13.60 ± 2.52^a^	15.03 ± 3.42^a^	10.57 ± 2.35^a^	13.37 ± 4.90^a^	12.90 ± 4.39^a^	15.53 ± 4.96^a^
Ca (%)	42.30 ± 1.82^a^	42.83 ± 1.62^a^	41.70 ± 2.38^a^	41.90 ± 1.15^a^	41.63 ± 1.17^a^	41.47 ± 0.35^a^	41.43 ± 0.47^a^	41.97 ± 1.31^a^
Mg (%)	41.47 ± 4.42^a^	40.77 ± 6.11^a^	41.30 ± 2.39^a^	40.17 ± 2.67^a^	44.63 ± 1.37^a^	41.90 ± 5.60^a^	43.17 ± 4.34^a^	37.63 ± 4.34^a^
Na (%)	2.80 ± 0.70^a^	3.10 ± 0.52^a^	3.37 ± 1.40^a^	2.93 ± 0.15^a^	3.12 ± 0.57^a^	3.27 ± 1.01^a^	2.47 ± 0.38^a^	2.87 ± 0.31^a^
N‐NO_3_ (mg/kg)	3.22 ± 0.50^bc^	2.72 ± 2.05^abc^	0.30 ± 0.22^a^	2.22 ± 2.16^abc^	3.44 ± 2.10^bc^	4.15 ± 0.65^c^	1.16 ± 0.83^ab^	1.75 ± 0.32^abc^
N‐NH_4_ (mg/kg)	0.72 ± 0.55^a^	0.78 ± 0.03^a^	0.58 ± 0.28^a^	0.42 ± 0.06^a^	0.78 ± 0.38^a^	0.47 ± 0.06^a^	0.63 ± 0.03^a^	0.60 ± 0.09^a^
Sand (%)	80.00 ± 1.00^a^	80.67 ± 1.53^a^	81.33 ± 2.08^a^	80.67 ± 1.53^a^	79.67 ± 1.53^a^	80.33 ± 2.08^a^	80.00 ± 1.00^a^	80.67 ± 0.58^a^
Silt (%)	2.33 ± 0.58^a^	2.33 ± 0.58^a^	2.00 ± 1.00^a^	2.00 ± 1.00^a^	1.67 ± 0.58^a^	2.00 ± 0.00^a^	4.67 ± 1.53^b^	7.33 ± 0.58^c^
Clay (%)	17.47 ± 1.16^bc^	17.00 ± 1.73^bc^	16.67 ± 1.16^bc^	16.33 ± 0.58^bc^	18.67 ± 1.16^c^	17.67 ± 2.08^bc^	15.33 ± 1.53^b^	12.00 ± 0.00^a^
OC (%)	0.48 ± 0.10^a^	0.47 ± 0.12^a^	0.40 ± 0.03^a^	0.40 ± 0.01^a^	0.40 ± 0.09^a^	0.37 ± 0.08^a^	0.40 ± 0.04^a^	0.46 ± 0.10^a^

*Note:* The alphabets within each group indicate significant differences (*p* < 0.05) between treatments, expressed as the means ± standard deviations.

*S value = sum of extractable Ca, Mg, K, and Na (c.mol(+)/kg)(me%). Soil after cowpea (G1 and G2), soil after soybean (G5 and G6), soil after maize (G7 and G8), and soil without precrop (G3 and G4).

Starting in February 2024, eight treatments were established with two cultivars of sorghum: “Avenger” and “NS55.” The treatments were as follows: sorghum “Avenger” following cowpea systems (G1), sorghum “NS55” following cowpea systems (G2), sorghum “Avenger” following soybean systems (G5), sorghum “NS55” following soybean systems (G6), sorghum “Avenger” following maize systems (G7), sorghum “NS55” following maize systems (G8), sorghum “Avenger” with no precrop or plant residues (G3), and sorghum “NS55” with no precrop or plant residues (G4). Each treatment was performed in triplicate, and crop management, including pesticide use, irrigation, and planting density, was standardized across all systems. The size of each field is 20.80 m^2^ (5.20 m × 4 m), with eight rows of plants per experimental field, and each field is separated by 1 m.

### 2.2 Collection of Soil Samples From All the Cropping Systems

To guarantee a stable environment for microbial communities and functional traits, we collected samples in June 2024, during the soft dough phase of the sorghum life cycle. We took 20–30 sorghum plants randomly from each field, shaken the lightly attached soil from the roots, and then collected the soils that were closely knitted with the sorghum rhizosphere. In total, we collected 24 rhizosphere soil samples from G1, G2, G5, G6, G7, G8, G3, and G4 (8 systems × 3 replicates). To remove root debris, the samples were sieved through 2 mm meshes and separated into two groups. One group was instantly transported to the laboratory in a box filled with ice and stored at −80°C for DNA extraction (Enagbonma and Babalola 2020), whereas the other group was stored at 4°C for soil analysis (Enagbonma et al. 2020).

### 2.3 Soil Analysis

Soils from the sorghum rhizosphere across each treatment were assessed to determine the physical and chemical parameters following standard procedures used at the Agricultural Research Council, Rustenburg, South Africa. After air drying, measurements were taken for available potassium (K), nitrate (N‐NO_3_, pH, available phosphorus (P), ammonium (N‐NH_4_), organic carbon (OC), sand, silt, clay, sodium (Na), *S, and magnesium (Mg) contents via methods outlined in previous studies (Ogundijo et al. 2015; Enagbonma and Babalola 2020, 2022).

### 2.4 Metagenomic Analysis and Gene Annotation

Total DNA was extracted from 0.5 g of rhizosphere soil per sample via the Nucleospin Soil Kit (Macherey‐Nagel, Germany) according to the manufacturer's instructions. To explore the structural and functional traits of the microbiome from sorghum rhizospheres, 24 DNA samples (representing 8 systems, each with 3 replicates) were chosen for shotgun metagenomic sequencing on the Illumina podium (NovaSeq. 6000), resulting in 150‐base‐pair paired‐end reads. For library preparation, 1 μg of genomic DNA was split into approximately 350 base pairs via a Covaris ultrasonic disruptor. The process involved PCR amplification, adapter ligation, purification, A‐tailing, and end repair. Fragment size and integrity were verified via AATI analysis. After confirming the insert size, Q‐PCR was utilized to determine the effective concentration of the library, which exceeded 3 nM for quality assurance. Once the quality requirements were met and based on the concentration, the libraries were pooled, and the target data were obtained before PE150 sequencing was performed. Fastp (version 0.23.4) (S. Chen 2023) was used for data preprocessing, filtering out reads containing adapter sequences, more than 10% ambiguous nucleotides, or more than 50% low‐quality bases (quality score < 5). To reduce host contamination, the cleaned reads were aligned against a host (with NCBI accession number ABXC00000000) reference catalog via Bowtie2 software (version 2.0) (Langmead and Salzberg 2012) with the following parameters: ‐X 400, ‐‐sensitive, ‐I 200, and ‐‐end‐to‐end. Assembly of the clean reads was performed via MEGAHIT software (version 1.0) (D. Li et al. 2016) with the ‐‐presets meta‐large parameter, whereas scaffolds containing “N” junctions were excluded to generate scaftigs (Lei et al. 2019). Open reading frames (ORFs) were predicted from scaftigs of at least 500 bp via MetaGeneMark (version 2.0) (Gemayel et al. 2022) with default parameters, and ORFs under 100 nt were excluded (Zeller et al. 2014). CD‐HIT software (version 3.1) was employed to remove redundant ORFs, producing a nonredundant (NR) gene database (Q. Chen et al. 2016). The quality control summary of the sequences can be found in Table S1. The clean data were aligned to this catalog via Bowtie2 (version 2.0), and genes with read counts ≤ 2 were excluded to finalize the gene catalog (unigenes) for subsequent analysis (Zeller et al. 2014). Gene abundance in each sample was determined on the basis of read counts and gene length (Villar et al. 2015). For taxonomic classification, DIAMOND software (version 0.7.9) (Buchfink et al. 2015) was employed to align unigene sequences with the Micro_NR database (which is a regularly updated database). This is a customized version of NCBI's NR database which includes viral, archaeal, fungal, and bacterial (K. Yu and Zhang 2013). MEGAN software (version 3.0) (Huson et al. 2007) was used for the taxonomic classification using the LCA algorithm). DIAMOND software (version 0.7.9) (Buchfink et al. 2015) was also employed to align unigene sequences with the functional databases (e.g., KEGG and eggNOG), via the BLASTP algorithm (version 2.x) with a parameter of 1e − 5 (W. Liu et al. 2021). The top BLAST hits were selected for further analysis (Buchfink et al. 2015). For each alignment, sequences with an *e* ≤ min. *e* value ∗ 10 was retained. From the LCA annotations and gene abundance data, the abundance of each taxonomic level (e.g., phylum and genus) in each sample was calculated. Species abundance was computed by summing the gene abundances annotated to that species (Karlsson et al. 2012). The number of genes per species was determined by counting genes with nonzero abundance annotated to that species. The abundance of the unigene in each sample was determined with the formula below:

$$G_{k}=\frac{T_{k}}{L_{k}}\cdot \frac{1}{\sum_{i=1}^{n}\frac{ri}{Li}},$$

where *G_k_* is the abundance of the unigene in the sample, *r* is the number of gene reads on alignment, *L* is the length of the gene, and *i* is the initial value (Adebayo et al. 2025). Additionally, relative abundances at different functional levels were computed by summing the relative abundances of genes associated with that function (Karlsson et al. 2012). A gene number table for each sample at each functional level was generated from the functional annotation and gene abundance data. The functional gene counts were determined on the basis of genes with nonzero abundance associated with that function. Further annotation of reads to nitrogen cycle‐related functional genes was performed via the NCycDB database (version 2.0) (Tu et al. 2019) at 100% identity, employing the DIAMOND algorithm (version 0.7.9) (Buchfink et al. 2015).

### 2.5 Statistical Analysis

One‐way analysis of variance (ANOVA) at a *p* < 0.05 was used to assess the differences in soil physicochemical parameters, diversity indices, phyla and genera abundances, and nitrogen functional genes among the treatments across the cropping systems. Shapiro‒Wilk tests in R software via the stats library were used for normalizing our data set, whereas Tukey means separation tests were used for post hoc comparisons at *p* < 0.05. To make samples comparable, all samples were rarefied with rarefy_even_depth() function in the phyloseq package using R software to the same sequencing depth before computing diversity indices (Natta et al. 2025). The alpha diversity (Shannon index) of the microbiome and functional genes was assessed via the phyloseq package, and visualizations were produced using ggplot2 (Suresh et al. 2025). The Kruskal–Wallis test was then used to test for significance. The beta diversity determined via non‐metric multidimensional scaling (NMDS) was used to evaluate the differences in microbiome composition between treatments. After standardizing and transforming (square root) our data sets, the community composition was evaluated via a Bray‒Curtis similarity matrix. This was performed with the vegan package in R, and the resulting ordinations were visualized using ggplot2 (Barnett et al. 2021). To estimate the statistical significance of crop rotation on community composition, permutational multivariate analysis of variance (PERMANOVA) with 9999 random permutations was employed. On the basis of the abundance table at each taxonomy and functional level, the relative abundance and the clustering heatmap were depicted (R ade4 package) (C. R. Rao 1964). On the basis of Bray‒Curtis data, we tested the correlations between soil physicochemical properties and the microbial community, and Spearman correlations were subsequently used for Mantel tests. Next, the correlation between microbial composition and soil physicochemical parameters was explored by employing the BEST routine, and the BIO‐ENV approach was employed to compute the minimum subset of soil physicochemical parameters that explained most of the variation in the microbial data set via Spearman's rank correlation. Analysis of dimension reduction (RDA) with a Monte Carlo permutation test was carried out with the vegan R package (Oksanen et al. 2016).

## 3 Results

### 3.1 Soil Physicochemical Parameters of the Various Cropping Systems

**Figure 1:**
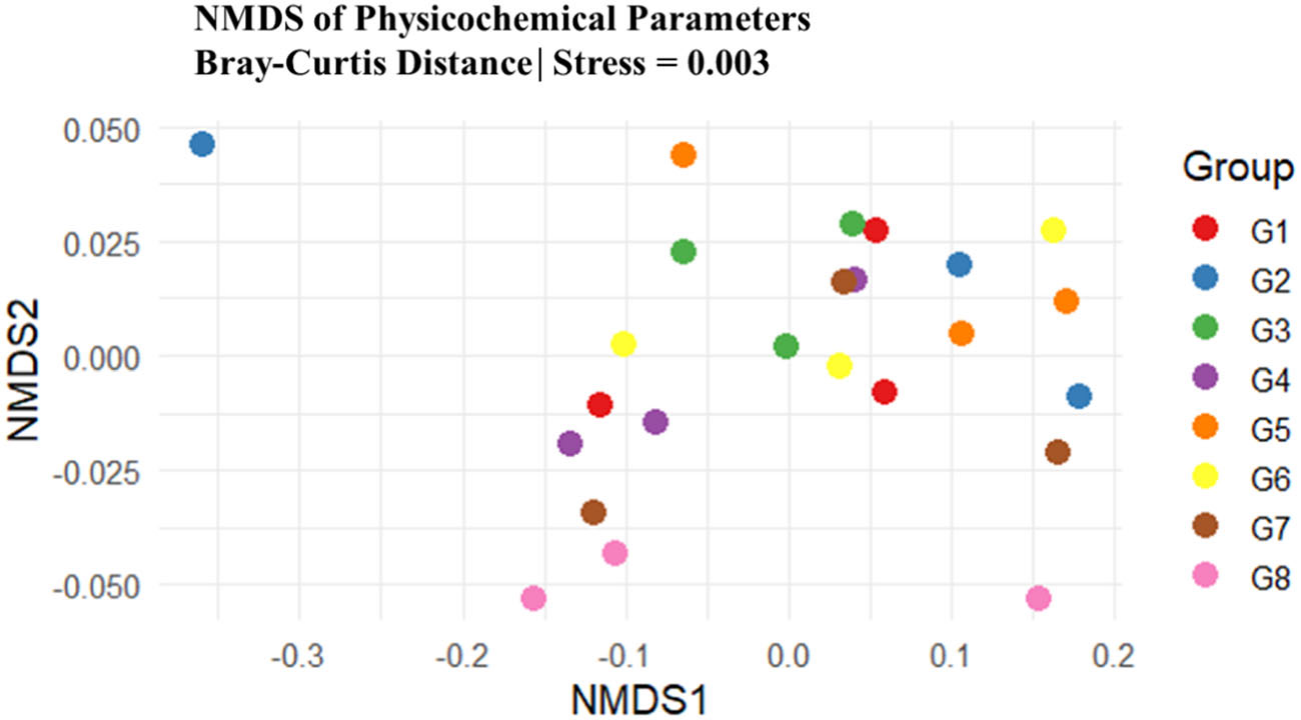
NMDS analysis of soil physicochemical parameters across various cropping systems on the basis of the Bray‒Curtis similarity matrix. The stress value (0.033) is appropriate when it is less than 0.20. Soil after cowpea (G1 and G2), soil after soybean (G5 and G6), soil after maize (G7 and G8), and soil without precrop (G3 and G4).

Initially, in this study, we evaluated the impact of the cropping system on soil physicochemical parameters (Table 1). Among both sorghum varieties (Avengers and NS55), the soil after soybean (G5 and G6) presented significantly greater N‐NO_3_ (one‐way ANOVA, *p* = 0.036) and clay contents (one‐way ANOVA, *p* = 0.035). They were followed by soil after cowpea (G1 and G2), sorghum without precrop (except in G3 in N‐NO_3_), and soil after the maize crop (G7 and G8). On the other hand, soil after maize crops (G7 and G8) was significantly more abundant (one‐way ANOVA, *p* = 0.041) in silt. This was followed by soil after cowpea (G1 and G2), sorghum without precropping (G3 and G4), and soil after cowpea (G1 and G2). Furthermore, in the rhizosphere soil after cowpea, relatively high values of N‐NO_4_, Ca, and OC were detected in relation to those in the other cropping systems. The NMDS examination revealed that the G8 (Pink) group forms a relatively tight cluster, mostly at the lower part of the plot. While in G2 (Blue), one point is far to the left, but the rest scatter a bit. This suggests partial distinctness. Furthermore, G1 (Red), G3 (Green), G4 (Purple), G5 (Orange), G6 (Yellow), and G7 (Brown) overlap heavily around the center, meaning their physicochemical parameters are very similar (Figure 1). PERMANOVA revealed that the soil physicochemical properties were significantly (*p* = 0.032, *R* ^2^ = 0.158 [≈ 16%], *F* = 4.14, stress = 0.033) influenced by the cropping system. RDA (Table S2) with a Monte Carlo permutation test further revealed that N‐NO_3_ (*p* = 0.008), clay (*p* = 0.004), and silt (*p* = 0.002) significantly contributed to these variations.

### 3.2 Influence of Cropping System on the Microbial Structure and Composition of the Sorghum Rhizosphere

**Figure 2:**
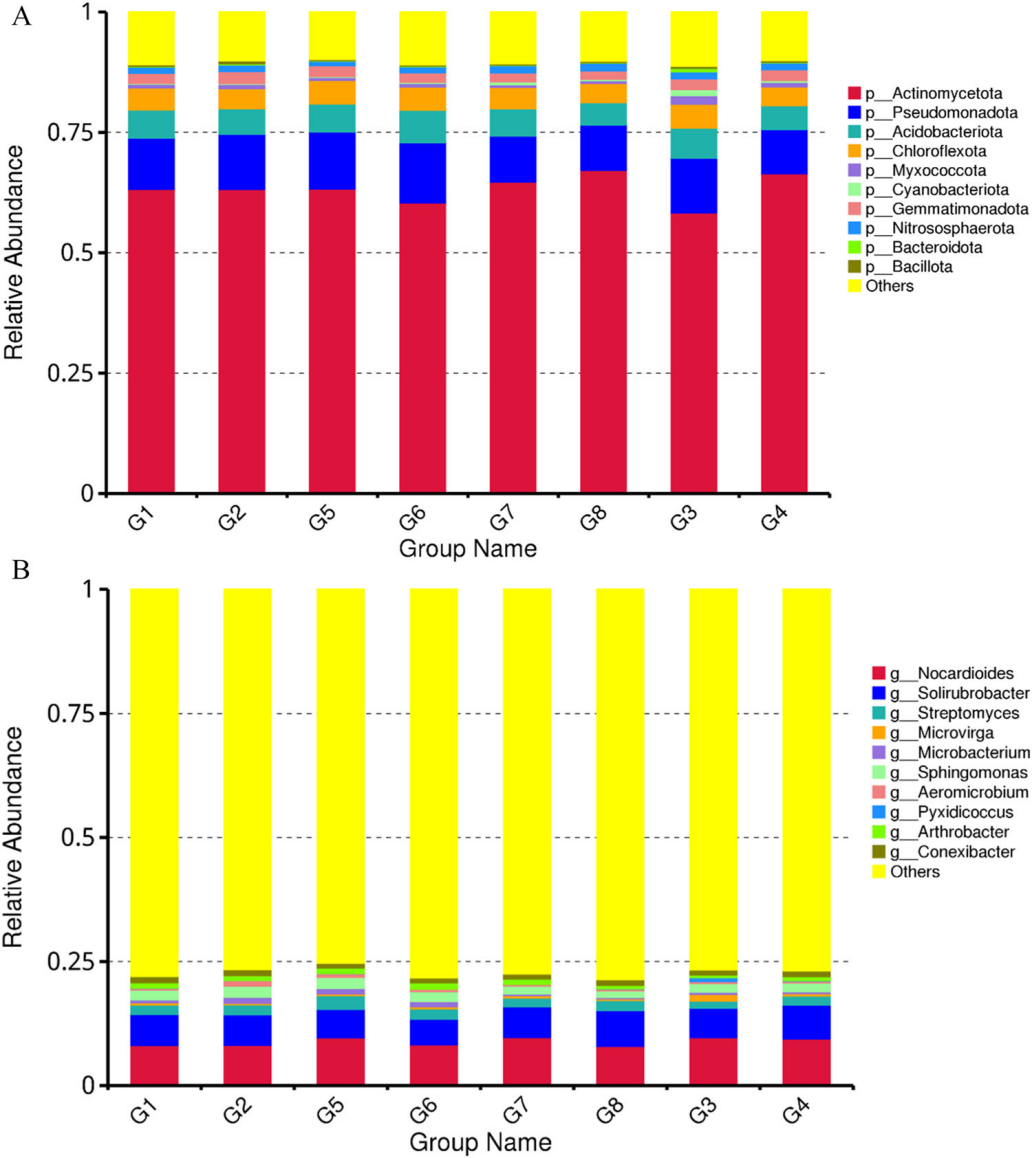
(A) The relative abundance of various microbial taxa (at the phylum level) across the cropping system. p stands for phylum. Soil after cowpea (G1 and G2), soil after soybean (G5 and G6), soil after maize (G7 and G8), and soil without precrop (G3 and G4). (B) The relative abundance of various microbial taxa (at the genus level) across the cropping system. g stands for genus. Soil after cowpea (G1 and G2), soil after soybean (G5 and G6), soil after maize (G7 and G8), and soil without precrop (G3 and G4).

Second, we examined how various precrops (cowpea, soybean, and maize) without precrops affected the soil microbiome structure. The rhizosphere microbiomes were allocated to 35 phyla (Figure S1), but in this study, 10 microbial phyla with the highest relative abundance across the samples were reported (Figure 2A). Actinomycetota was the most abundant bacteria across the cropping system and was highest in the soil after the maize crops (G7 and G8). This was followed by soil after soybean crops (G5 and G6), soil after cowpea crops (G1 and G2), and sorghum without precrops (G3 and G4). On the other hand, Pseudomonadota predominated in soil after soybean crops (G5 and G6), followed by soil after cowpea crops (G1 and G2), sorghum without precrops (G3 and G4), and soil after maize crops (G7 and G8). At the genus level, *Nocardioides* and *Solirubrobacter* predominated in the soil after maize crops (G7 and G8), whereas *Streptomyces* predominated in the soil after soybean crops (G5 and G6) (Figure 2B). Despite the variation observed, the impact of crop rotation on the microbial abundance categorized at the phylum (one‐way ANOVA, *p* = 0.61) and genus (one‐way ANOVA, *p* = 0.47) levels was not significant according to one‐way analysis.

### 3.3 Influence of Cropping System on the Microbiome Diversity (Alpha and Beta) of the Sorghum Rhizosphere

**Figure 3:**
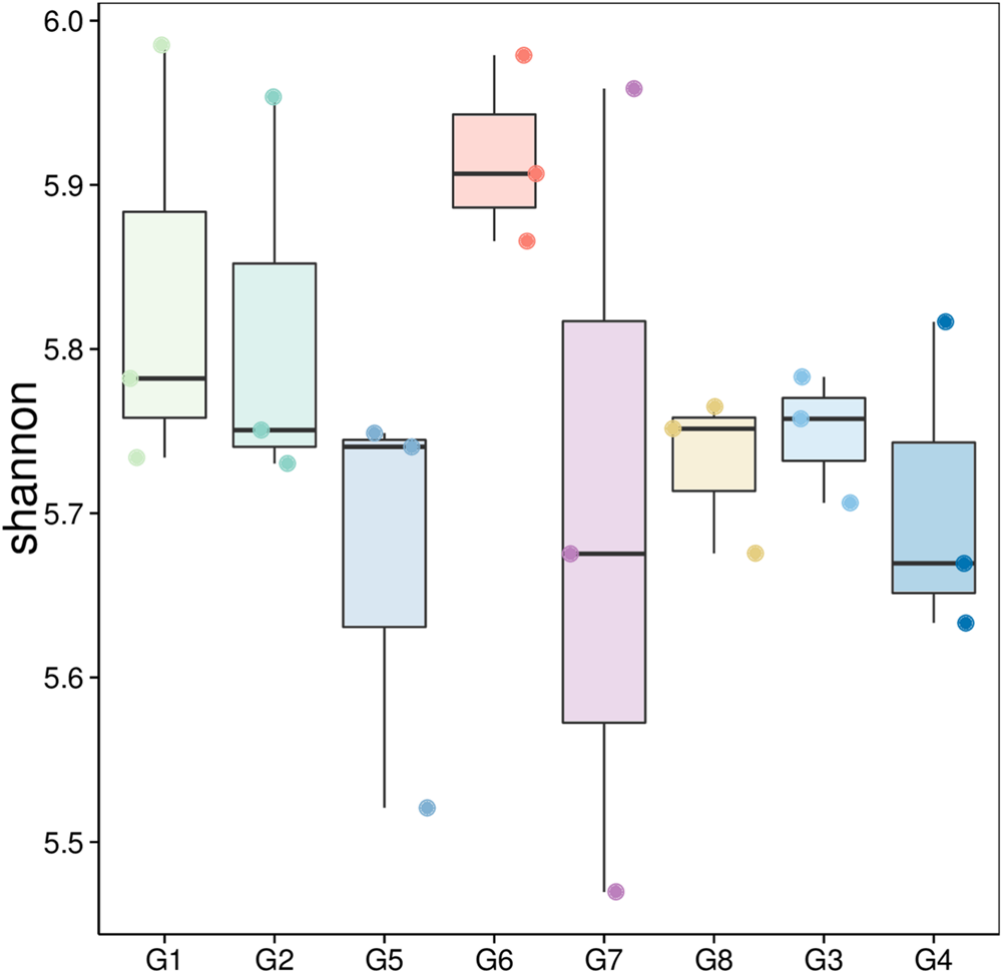
Box plot depicting the Shannon (alpha) diversity indices for various cropping systems, for the microbiome. Soil after cowpea (G1 and G2), soil after soybean (G5 and G6), soil after maize (G7 and G8), and soil without precrop (G3 and G4).

**Figure 4:**
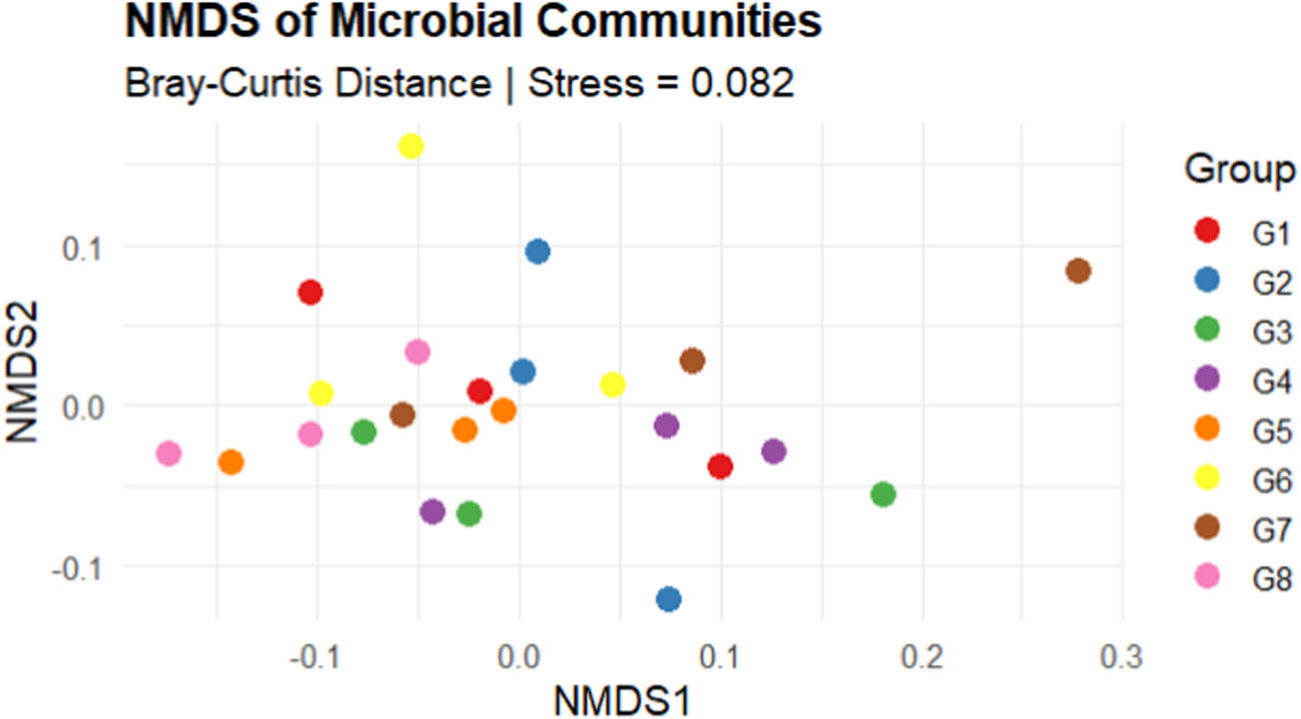
NMDS analysis of the microbial community across various cropping systems on the basis of the Bray‒Curtis similarity matrix. The stress value (0.082) is appropriate when it is less than 0.20. Soil after cowpea (G1 and G2), soil after soybean (G5 and G6), soil after maize (G7 and G8), and soil without precrop (G3 and G4).

The alpha diversity (Shannon) index differed significantly among the cropping systems (Figure 3). Crop rotation type significantly (Kruskal–Wallis test, *p* = 0.032) differentiated the microbial diversity in the soil. The soil after cowpea crops (G1 and G2) presented a relatively high median, indicating relatively high diversity within these samples compared with the soil after soybean crops (G5 and G6). The soil after maize crops (G7 and G8) was characterized by a median diversity index lower than that of G1, G2, and G5 but higher than that of sorghum without crops (G3 and G4). The spread (interquartile range) in G7 is notably large, indicating significant variability among the samples. Sorghum without precrops (G3 and G4) presented the lowest median values, indicating lower diversity. G4 displays considerable variability, as suggested by the wider box (Figure 3). The beta diversity depicted by the NMDS plot (Figure 4) shows that 39% of the variation in our microbial community data was explained by the grouping variable. PERMANOVA revealed that microbial communities were significantly (*p* = 0.001, *R* ^2^ = 0.39106 [≈ 39%], stress = 0.082, *F* = 8.549) influenced by the cropping system.

### 3.4 Cropping System Influences the Functional Gene Diversity of the Microbiome in the Sorghum Rhizosphere

**Figure 5:**
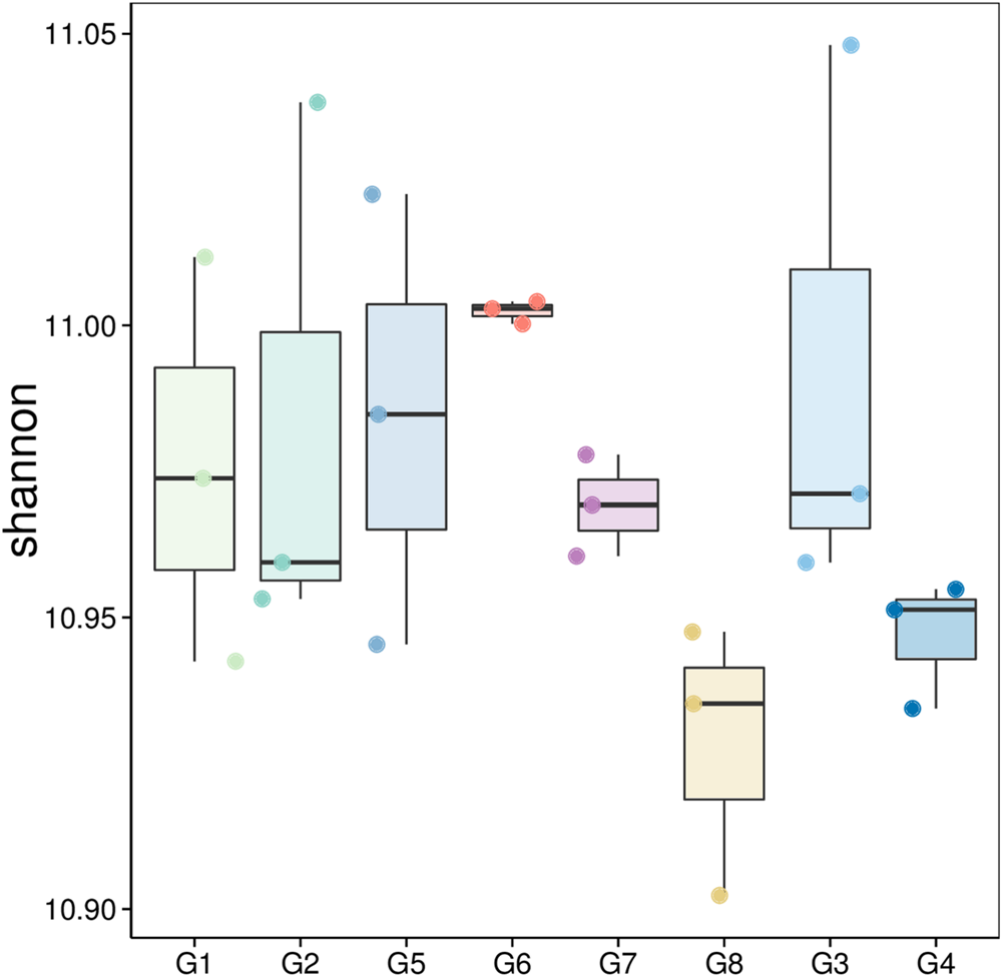
Box plot displaying the Shannon diversity indices for various cropping systems for the functional genes. Soil after cowpea (G1 and G2), soil after soybean (G5 and G6), soil after maize (G7 and G8), and soil without precrop (G3 and G4).

**Figure 6:**
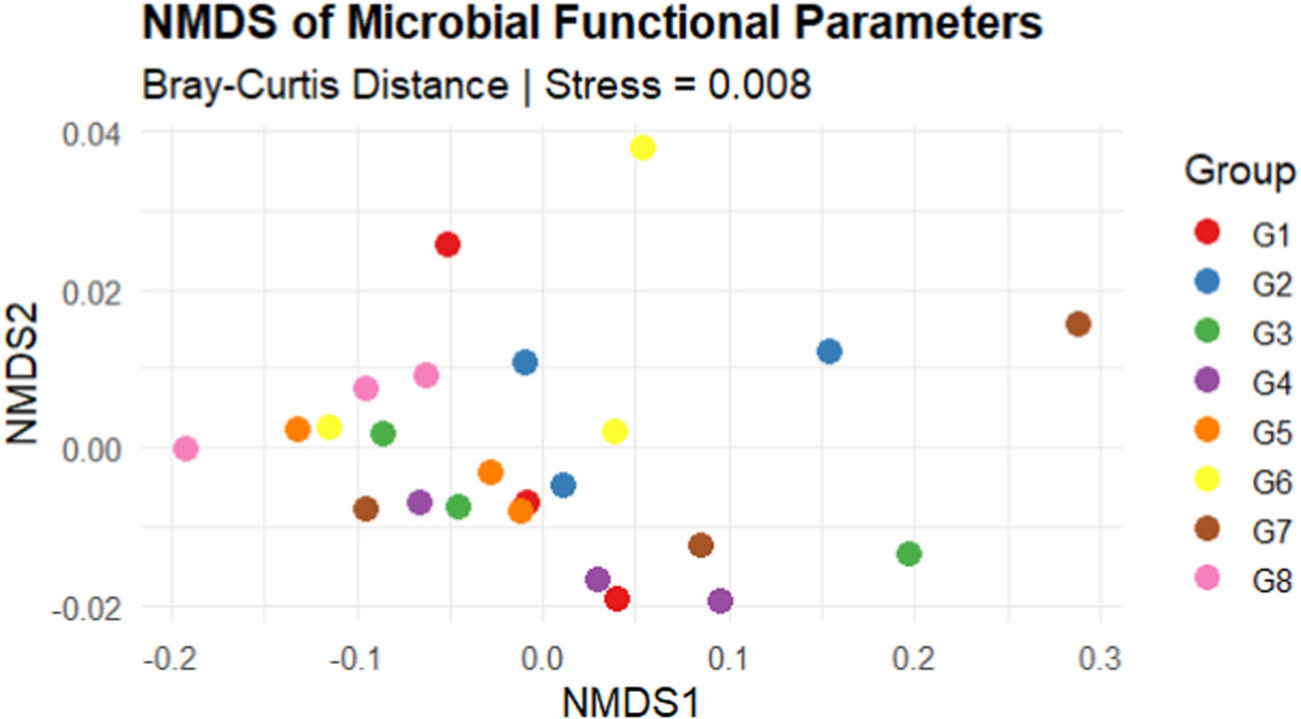
NMDS analysis of microbial functional diversity across various cropping systems on the basis of the Bray‒Curtis similarity matrix. The stress value (0.008) is appropriate when it is less than 0.20. Soil after cowpea (G1 and G2), soil after soybean (G5 and G6), soil after maize (G7 and G8), and soil without precrop (G3 and G4).

The box plot shows that the Shannon diversity indices differ significantly (Kruskal–Wallis test, *p* < 0.030) among the cropping systems (Figure 5). The functional gene diversity in the soil after soybean cropping (G5 and G6) was significantly greater than that in the soil after cowpea cropping (G1 and G2). Compared with those in the other groups, the functional gene diversity in the soil after maize cropping (G7 and G8) was noticeably lower. In sorghum without precrops, G3 had moderate functional gene diversity with lower variability, whereas G4 had a tighter distribution, indicating that the Shannon diversity in this group was more consistent with lower variability. The NMDS plot (Figure 6) shows that 90.2% of the variation in our microbial functional profile was explained by the grouping variable. PERMANOVA revealed that the microbial functional profile was significantly (*p* = 0.001, *R* ^2^ = 0.9021 [≈ 90%], stress = 0.008, *F* = 202.73) influenced by cropping system. This shows that their functional categories (reads to different functional groups depicted by the eggNOG and KEGG databases have been reported in Figures S2 and S3) are different.

### 3.5 Microbial Functional Genes Involved in Nitrogen Cycling

**Figure 7:**
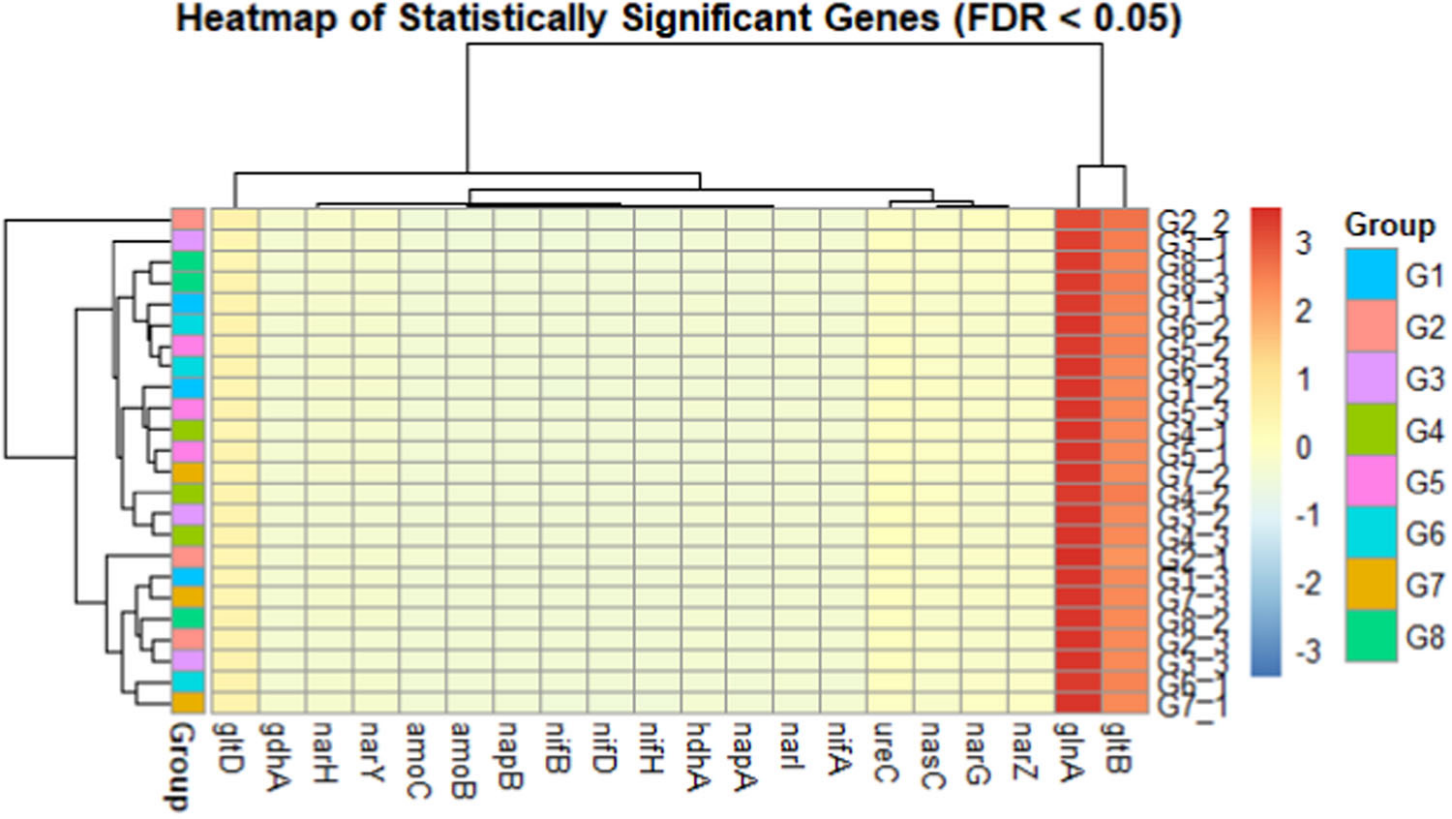
Distribution of functional genes involved in nitrogen cycling across the cropping system. The color bar on the right indicates relative abundance, where red represents high relative abundance and blue signifies low relative abundance. Soil after cowpea (G1 and G2), soil after soybean (G5 and G6), soil after maize (G7 and G8), and soil without precrop (G3 and G4).

In this study, 30 microbial functional genes encoding nitrogen fixation, nitrification, assimilation, and amplification pathways were identified across the different groups (Figure S4). However, 20 of the 30 microbial functional genes were significantly different (FDR < 0.05) (Figure 7). The soil after cowpea crops (G1 and G2) was dominated by *amoC*, *narH, gltB*, and *glnA* (*glnA* and *gltB* are functional genes involved in assimilatory nitrogen metabolism), whereas the soil after soybean crops (G5 and G6) was dominated by *ureC* and *napA* (*napA* is a gene involved in dissimilatory nitrogen metabolism). On the other hand, soil after maize crops (G7 and G8) predominated *glnA*, *gltB, narZ*, and *narH*, whereas sorghum without precrops (G3 and G4) predominated *glnA*, *narZ*, and *ureC*.

### 3.6 Ecological Filters of Sorghum Rhizosphere Microbiome Genes Across Different Cropping Systems

**Figure 8:**
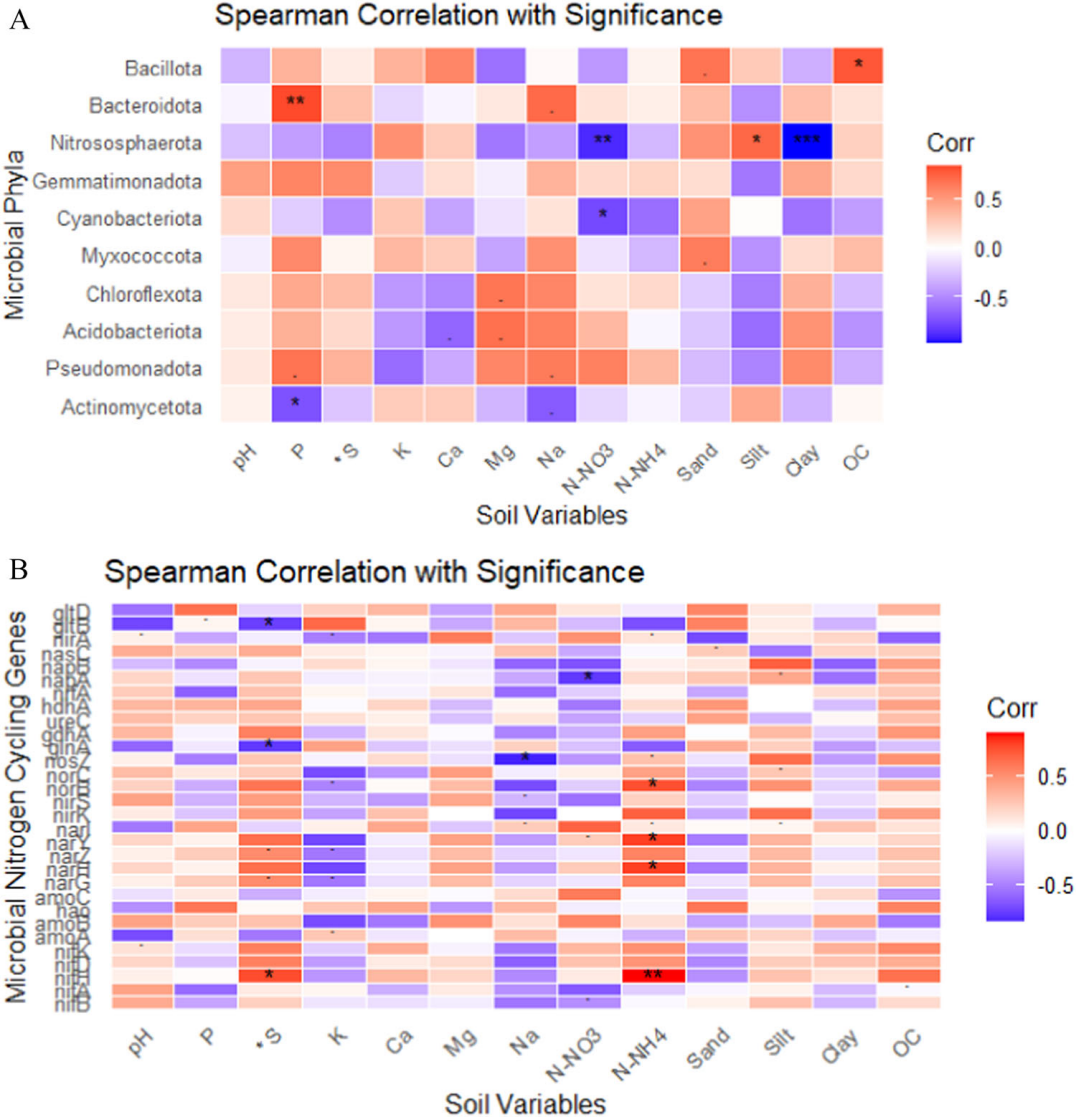
(A) Correlation analysis between the physicochemical parameters of the soil and the microbial community and their nitrogen‐cycling genes. (B) Correlation analysis between the physicochemical parameters of the soil and the microbial nitrogen‐cycling genes.

Our final objective was to assess the relationships between the physicochemical parameters of the soil and the microbial community and their nitrogen‐cycling genes. Many significant correlations were observed between the soil physicochemical properties and the microbial community (Figure 8A). Soil nutrients such as P, OC, and N‐NO_3_ significantly influence microbial distributions, while soil texture and structure (clay and silt) also play significant roles. For example, Bacillota was positively correlated with OC (*p* < 0.05), Bacteroidota was positively correlated with P (*p* < 0.01), Nitrososphaerota was negatively correlated with N‐NO_3_ (*p* < 0.01) and clay (*p* < 0.001) and positively correlated with silt (*p* < 0.05), Cyanobacteriota was negatively correlated with N‐NO_3_ (*p* < 0.05), and Actinomycetota was negatively correlated with *p* < 0.05). Among the nitrogen‐cycling genes, *norB* (*p* < 0.05), *narl* (*p* < 0.05), *narH* (*p* < 0.05), and *nifH* (*p* < 0.01) were positively correlated with N‐NO_4,_ and *napA* was negatively correlated with N‐NO_3_ (*p* < 0.05); *nosZ* was negatively correlated with Na (*p* < 0.05), *glnA* (*p* < 0.05), and *gltB* (*p* < 0.05) was negatively correlated with *S and *nifH* (*p* < 0.05) and positively correlated with *S. BEST analysis of 13 environmental variables revealed that *S, Na, N‐NH_4_, N‐NH_3_, and P, and clay were most strongly correlated with microbial community patterns and explained approximately 36% of the variation in microbial community structure (based on rank relationships).

## 4 Discussion

In this study, we evaluated the effects of the application of cowpea, soybean, and maize in a cropping system (with the return of crop residue to the soil) on microbial communities and their functional nitrogen genes during the soft dough phase while considering the physicochemical parameters of the soil. Crop rotation significantly impacted the soil physicochemical and microbial parameters, depending on the factor examined.

### 4.1 Variation in Soil Physicochemical Properties Under Precrops

The findings revealed that N‐NO_3,_ clay, and silt contents changed significantly depending on the crop sequence. Soil after soybean significantly influenced the N‐NO_3_ and clay contents, whereas soil after maize significantly influenced the silt content. Although the soil after cowpea cropping presented higher values of N‐NO_4_, Ca, and OC than did the other cropping systems, these differences were not significant. The high value of N‐NO_3_ content in the soil after legume (soybean and cowpea) cultivation compared with that in the soil after maize and sorghum cultivation without precropping can be attributed in part to the relatively high N content in the soybean and cowpea residues. Additionally, nitrogen from symbiotic fixation, the discharge of nitrogen from nodules and roots, and nitrogen rhizodeposition occur (S. Li et al. 2021; Sebetha et al. 2015). The high N‒NO_3_ content in soybean compared with cowpea could be related to the high amount of nitrogen symbiotically fixed and positive soil N balance in soybean (Gx 1448‐‐2E) compared with cowpea (IT96D‐724), as reported by Yusuf et al. (2008). OC is crucial for soil fertility, carbon dynamics, and microbial communities, and its high content in soil after cowpea compared with that in soil after maize could be due to carbon rhizodeposition (Zang et al. 2018; Becker et al. 2024). OC and N‐NO_3_ contributed strongly to the microbial community in our study. This finding is in line with those of Cui et al. (2016) and Hou et al. (2023). Our findings support other studies revealing a greater beneficial impact of legumes than of cereal residue on increasing N cycling in agroecosystems. A correlation between soybean and clay contents has been reported by Cox et al. (2003), where clay was reported to be a common factor affecting soybean yields. This could explain why the clay content was greater in the soil after soybean and cowpea cultivation than in the soil after maize and sorghum cultivation without precrop cultivation. The higher clay and lower silt contents in the sorghum rhizosphere after cowpea and soybean precropping than in the maize rhizosphere are likely due to the finer roots, nitrogen‐rich residues, and greater microbial activity of the former, which improve the soil structure and help clay particles stay in place. These conditions also allow silt to move more easily through the soil. Maize, with its relatively thick roots and relatively slow residue breakdown, has a relatively small effect on the soil texture, leading to relatively little clay and more silt in the rhizosphere (Farouq et al. 2022; Kombat et al. 2024; Qi et al. 2024).

### 4.2 Precropping in a Crop Rotation‐Shaped Microbiome in the Sorghum Rhizosphere

The high N‐NO_3_, Silt, N‐NO_4_, Ca, and OC contents in the soil after soybean and cowpea were precropped compared with those in the soil before cropping and sorghum cultivation without precropping could be explained by a relatively high decomposition rate, which is associated with high microbiological activity owing to root exudates (Moreno et al. 2021). Shannon index, NMDS, and PERMANOVAs revealed a significant effect, indicating that crop rotation, by altering soil parameters, influenced the microbial community composition. These findings correspond with earlier studies examining the effects of the integration of soybean and cowpea in crop rotation on soil microorganisms (Babalola and Enagbonma 2025b; Araujo et al. 2023). With the aid of a metagenomic approach, H. Yu et al. (2021) detected variations in the microbial compositions of soil microbiomes in legume rotation versus nonlegume rotation. D. Rao et al. (2021) also reported that the activities of soil microbiomes were positively dependent on the C/N ratio in soil with integrated organic materials because of competition among microbes and crops for nitrogen. S. Chen et al. (2022) and C. Liu et al. (2025) reported that crop rotation drives microbial community assembly by altering the quantity, quality, and diversity of inputs into the soil, and this in turn shapes selective pressures on microorganisms. Each crop species releases distinct root exudates, residues, and secondary metabolites that act as carbon and nutrient sources, or sometimes as inhibitory compounds (Moore et al. 2014; Enagbonma et al. 2025). These differences create niche differentiation that allows specific microbial taxa with matching metabolic traits to proliferate (Hale et al. 2018). Our research revealed that the incorporation of soybean and cowpea in crop rotation increased the relative abundances of Pseudomonadota and *Streptomyces*. This may have a considerable effect on soil conditions because these bacteria release bioactive compounds with insecticide, antimicrobial, and plant growth‐promoting traits and multidrug resistance (Khoshru et al. 2025; Imade et al. 2024; Al‐Quwaie 2024). For example, *Streptomyces* break down complex organic matter such as cellulose and chitin, thereby enhancing carbon cycling and soil fertility (Ugochukwu and Okorie 2020). They are also prolific producers of antibiotics, which enable them to suppress plant pathogens and act as effective plant growth‐promoting rhizobacteria through mechanisms such as siderophore production and phytohormone synthesis (Donald et al. 2022). AbdElgawad et al. (2020) reported that the soil rhizosphere possesses a high abundance of Actinomycetes. This could be explained by the fact that, compared with the other phyla, Actinomycetota was more abundant in all the cropping systems in our study. By increasing nitrogen availability and metabolism, Actinomycetes improve the soil rhizosphere seed quality and productivity of legumes. Actinomycetes promote nitrogen accessibility in legume seeds and tissues as well as in soil, which prompts the action of significant nitrogen‐metabolizing enzymes, such as nitrate reductase, glutamate synthase, and glutamine synthetase (AbdElgawad et al. 2020). These findings indicate that the emergent roots of soybean and corn impact the rhizosphere actinomycete structure. Abraham and Herr (1964) reported that the abundance of Actinomycete was significantly greater in the corn rhizosphere than in the soybean rhizosphere. This finding was in line with our finding that Actinomycetota predominates in soil after maize crops when related to other precorps. In contrast, some possibly useful rhizosphere microbes (e.g., *Pyxidicoccus*, *Microbacterium*, and *Microvirga*) can be reduced by incorporating precrops in rotation; for example, predatory *Pyxidicoccus* (a genus from Myxobacteria) and various antimicrobial compounds position them as potential solutions that can hinder biofilm formation and microbial growth (Arakal 2025). Cordovez et al. (2018) reported that root‐associated *Microbacterium* strains release volatiles that increase the root architecture and root and shoot biomass of *Arabidopsis*. Furthermore, the microbiome structure was significantly shaped by soil physical and chemical parameters. Therefore, the positive impact of legume precrops on soil microbiomes could partly result from their increasing residual soil water content and impact on aggregate stabilization and soil structure (Siczek et al. 2024).

### 4.3 Precropping in Crop Rotation Influenced Microbial Functional Genes in the Sorghum Rhizosphere

The functional gene analysis further demonstrated the impact of cropping patterns on the rhizosphere microbiome. The diversity of nitrogen functional genes in soil reflects the ability of the soil microbiome to perform many metabolic activities and react to ecological changes, which is influential for preserving soil function and health. Soils preceded by legumes are richer in nitrogen‐cycling functional genes, such as *amoC, narH, gltB, glnA, ureC, napA*, and *napA*. Understanding the roles of these genes provides insight into the complex processes of the nitrogen cycle, highlighting how microorganisms contribute to nitrogen transformation in various agroenvironments (Enagbonma and Babalola 2023). For example, ammonia monooxygenase (*amoC*) catalyzes the oxidation of ammonia (NH₃) to hydroxylamine (NH₂OH), which is a key step in the conversion of ammonia to nitrite (NO₂⁻) during nitrification (Wendeborn 2020). The *narH* is part of the *narGHI* complex, which reduces nitrate (NO_3_⁻) to nitrite (NO₂⁻). This step is common to both the DNRA and denitrification pathways. This gene influences nitrogen (N) use efficiency and crop production in arid farmlands. The *glnA* incorporates ammonium (NH₄⁺) into glutamine and maintains nitrogen homeostasis, critical under low‐nitrogen conditions (Zhou et al. 2022). Our research revealed that, compared with G7, G8, G3 and G4, G1, G2, G5, and G6 significantly increased the Shannon index of important functional genes, indicating that introducing legumes (soybean and cowpea) into crop rotations increased the functional potential of nitrogen cycling in the cropping system, whereas incorporating maize into rotation had a greater impact on the functional potential of nitrogen cycling in the ecosystem. The resilience and stability of soil ecosystems are influenced by soil functional gene diversity, as it can increase functional redundancy and prevent instability. Particularly in semiarid zones, integrating legumes into crop rotations can significantly increase the risk resistance of soil environments. We predict that the core explanations for the variation in fundamental gene diversity among the diverse cropping systems include the following: (i) At the developmental stage, legume associations with some bacteria, such as *Actinobacteria*, cause an increase in the abundance of specific genes (Greenlon et al. 2019); (ii) residues of legume precrops have a reduced C/N, making decomposition easier and facilitating the decomposition of soil minerals for microorganisms (Nguyen et al. 2016); and in applied farming management, the amount of crop residue in maize crops is greater than that in legume crops because the nitrogen application rate for legume crops is only 1/6 of that for maize crops. This causes a deficiency in soil nitrogen, resulting in a competitive correlation between crops and microorganisms, with crops being disadvantaged in their association (X.‐X. Wang et al. 2018; Hodge and Fitter 2010).

### 4.4 Relationships Between the Soil Environmental Factors and the Microbial Community

The distribution of microbial communities and nitrogen‐cycling genes is heavily influenced by edaphic factors (Forbes et al. 2009). The BEST analysis in our study identified nitrate nitrogen (N‐NO_3_), clay, and phosphorus (P) as key drivers of microbial diversity and composition in legume‐preceded sorghum soils. In contrast, *S, Na, and N‐NH₄ were most strongly correlated with microbial nitrogen‐cycling genes and explained approximately 36% of the variation in the microbial community structure. This highlights the importance of soil nutrient variables in shaping microbial communities in the rhizosphere, especially in soils enriched with legume residues (Kelly et al. 2022). These findings also indicate that soil texture and ammonium availability play crucial roles in maintaining nitrogen functionality in sorghum soils, particularly in legume‐preceded systems where nitrogen fixation is a key process. Interestingly, this study also revealed that N‐NH_4_ is the dominant factor shaping microbial functional traits in soils preceded by legumes, whereas silt plays a more prominent role in soils without legumes. This suggests that legume residues may increase ammonium availability (Azam et al. 1995), further supporting the growth of nitrogen‐fixing microorganisms (Franche et al. 2009), whereas in nonleguminous soils, soil texture, particularly silt, becomes a more important determinant of nitrogen‐cycling processes. Additionally, the observed influence of edaphic factors such as soil texture and nitrogen forms (N‐NO_3_ and N‐NH_4_) on microbial communities and nitrogen‐cycling genes emphasizes the importance of site‐specific soil management practices. Tailoring crop rotation and soil management to optimize these factors can further increase microbial functionality and improve nitrogen use efficiency in agricultural systems.

## 5 Conclusion

With a metagenomic sequencing approach, we made a substantial effort to determine the impact of sorghum cultivated in soils previously grown with cowpea, soybean, and maize on the rhizosphere microbiome and soil nitrogen cycling. Our research findings highlight the contributions of precropping of legumes in rotation to the modulation of rhizosphere microbiomes under a second sequential cereal crop. Cowpea and soybean rotation have a greater beneficial impact than does maize or sorghum cultivation without precrops on nutrient cycling, as shown by the values of available nutrients, which could increase crop productivity. Precropping affects both microbial communities, and these microbiomes vary in response to crop rotation. We further revealed that the diversity indices of microbiomes and nitrogen functional genes were influenced by crop rotation. Furthermore, precrops in rotation increased the abundance of some plant growth‐promoting genera, and our study broadens our understanding of the response of soil microbiomes under different precrops and helps with the precise prediction of shifts in the soil microbial community composition and function when crop rotation is established.

## Author Contributions

**Ben Jesuorsemwen Enagbonma:** writing – original draft, formal analysis, data curation, investigation. **David Mxolisi Modise:** supervision, project administration. **Olubukola Oluranti Babalola:** conceptualization, supervision, project administration, writing – review and editing, resources.

## Ethics Statement

The authors have nothing to report.

## Consent

The authors have nothing to report.

## Conflicts of Interest

The authors declare no conflicts of interest.

## Supporting information

MicrobiologyOpen supplimentary_revised.

## Data Availability

The sequences obtained from this study have been submitted to the Sequence Read Archive (SRA) at the National Center for Biotechnology Information (NCBI) under the assigned bioproject numbers PRJNA1166242 (G1), PRJNA1168160 (G2), PRJNA1166458 (G5), PRJNA1166463 (G6), PRJNA1166623 (G7), PRJNA1166627 (G8), PRJNA1166630 (G3), and PRJNA1166631 (G4).

## References

[mbo370085-bib-0001] AbdElgawad, H. , W. Abuelsoud , M. M. Y. Madany , et al. 2020. “Actinomycetes Enrich Soil Rhizosphere and Improve Seed Quality as Well as Productivity of Legumes by Boosting Nitrogen Availability and Metabolism.” Biomolecules 10, no. 12: 1675. 33333896 10.3390/biom10121675 PMC7765327

[mbo370085-bib-0002] Abraham, T. A. , and L. J. Herr . 1964. “Activity of Actinomycetes From Rhizosphere and Nonrhizosphere Soils of Corn and Soybean in Four Physiological Tests.” Canadian Journal of Microbiology 10, no. 2: 281–285. 10.1139/m64-036.

[mbo370085-bib-0003] Adebayo, A. A. , B. J. Enagbonma , and O. O. Babalola . 2025. “Comparative Metagenomics on Community Structure and Diversity of Rhizomicrobiome Associated With Monoculture and Soybean Precedent Carrot.” Scientific Reports 15, no. 1: 28161. 40750823 10.1038/s41598-025-13605-z PMC12317137

[mbo370085-bib-0004] Akhtar, S. , S. Bashir , S. Khan , et al. 2020. “Integrated Usage of Synthetic and Bio‐Fertilizers: An Environment Friendly Approach to Improve the Productivity of Sorghum.” Cereal Research Communications 48: 247–253.

[mbo370085-bib-0005] Allie, G. , S. Karteh , M. S. E. W. Ngegba , M. J. Sandy , and D. M. Ibrahim‐Sayo . 2025. “Nutritional Quality and Sensory Profile of “Oleleh” Produced From Cowpea (*Vigna unguiculata*) and Soybean (*Glycine max*) Composite.” Journal of Underutilized Legumes 7, no. 1: 48–56.

[mbo370085-bib-0006] Al‐Quwaie, D. A. 2024. “The Role of *Streptomyces* Species in Controlling Plant Diseases: A Comprehensive Review.” Australasian Plant Pathology 53, no. 1: 1–14.

[mbo370085-bib-0007] Amoo, A. E. , B. J. Enagbonma , A. S. Ayangbenro , and O. O. Babalola . 2021. “Biofertilizer: An ECo‐Friendly Approach for Sustainable Crop Production.” In Food Security and Safety: African Perspectives, edited by O. O. Babalola , 647–669. Springer.

[mbo370085-bib-0008] Ananda, G. K. S. , H. Myrans , S. L. Norton , R. Gleadow , A. Furtado , and R. J. Henry . 2020. “Wild Sorghum as a Promising Resource for Crop Improvement.” Frontiers in Plant Science 11: 1108. 32765575 10.3389/fpls.2020.01108 PMC7380247

[mbo370085-bib-0009] Arakal, B. S. 2025. Exploring the Antimicrobial Potential of Myxobacteria. Cardiff Metropolitan University.

[mbo370085-bib-0010] Araujo, A. S. F. , A. R. L. Miranda , A. P. A. Pereira , et al. 2023. “Microbial Communities in the Rhizosphere of Maize and Cowpea Respond Differently to Chromium Contamination.” Chemosphere 313: 137417. 36460149 10.1016/j.chemosphere.2022.137417

[mbo370085-bib-0011] Azam, F. , R. L. Mulvaney , and F. W. Simmons . 1995. “Effects of Ammonium and Nitrate on Mineralization of Nitrogen From Leguminous Residues.” Biology and Fertility of Soils 20: 49–52.

[mbo370085-bib-0012] Babalola, O. O. , A. A. Adebayo , and B. J. Enagbonma . 2025. “Shotgun Metagenomics Dataset of the Core Rhizo‐Microbiome of Monoculture and Soybean‐Precedent Carrot.” BMC Genomic Data 26: 26. 40221653 10.1186/s12863-025-01320-7 PMC11993964

[mbo370085-bib-0013] Babalola, O. O. , and B. J. Enagbonma . 2024. “Dataset of Amplicon Metagenomic Assessment of Barley Rhizosphere Bacteria Under Different Fertilization Regimes.” Data in Brief 52: 109920. 38186742 10.1016/j.dib.2023.109920 PMC10770713

[mbo370085-bib-0014] Babalola, O. O. , and B. J. Enagbonma . 2025a. “Dataset of Shotgun Metagenomic Evaluation of *Sorghum bicolor* Rhizosphere Microbiome in Soils Preceded by *Glycine max* .” Data in Brief 58: 111270. 39906131 10.1016/j.dib.2025.111270 PMC11791250

[mbo370085-bib-0015] Babalola, O. O. , and B. J. Enagbonma . 2025b. “Dataset of Shotgun Metagenomic Evaluation of *Sorghum bicolor* Rhizosphere Microbiome in Soils Preceded by *Glycine max* .” Data in Brief 58: 111270. 39906131 10.1016/j.dib.2025.111270 PMC11791250

[mbo370085-bib-0016] Bakari, H. , Djomdi , Z. F. Ruben , et al. 2023. “Sorghum (*Sorghum bicolor* L. Moench) and Its Main Parts (By‐Products) as Promising Sustainable Sources of Value‐Added Ingredients.” Waste and Biomass Valorization 14, no. 4: 1023–1044.

[mbo370085-bib-0017] Barnett, D. , I. Arts , and J. Penders . 2021. “microViz: An R Package for Microbiome Data Visualization and Statistics.” Journal of Open Source Software 6, no. 63: 3201.

[mbo370085-bib-0018] Becker, J. N. , J. Grozinger , A. Sarkar , B. Reinhold‐Hurek , and A. Eschenbach . 2024. “Effects of Cowpea (*Vigna unguiculata*) Inoculation on Nodule Development and Rhizosphere Carbon and Nitrogen Content Under Simulated Drought.” Plant and Soil 500, no. 1: 33–51.

[mbo370085-bib-0019] Buchfink, B. , C. Xie , and D. H. Huson . 2015. “Fast and Sensitive Protein Alignment Using DIAMOND.” Nature Methods 12, no. 1: 59–60. 25402007 10.1038/nmeth.3176

[mbo370085-bib-0020] Chen, S. 2023. “Ultrafast One‐Pass FASTQ Data Preprocessing, Quality Control, and Deduplication Using Fastp.” Imeta 2, no. 2: e107. 38868435 10.1002/imt2.107 PMC10989850

[mbo370085-bib-0021] Chen, Q. , Y. Wan , Y. Lei , J. Zobel , and K. Verspoor . 2016. “Evaluation of CD‐HIT for Constructing Non‐Redundant Databases.” In 2016 IEEE International Conference on Bioinformatics and Biomedicine (BIBM), 703–706. IEEE. 10.1109/BIBM.2016.7822604.

[mbo370085-bib-0022] Chen, S. , L. Wang , J. Gao , et al. 2022. “Agricultural Management Drive Bacterial Community Assembly in Different Compartments of Soybean Soil–Plant Continuum.” Frontiers in Microbiology 13: 2022. 10.3389/fmicb.2022.868307. PMC9114711 35602087

[mbo370085-bib-0023] Cordovez, V. , S. Schop , K. Hordijk , et al. 2018. “Priming of Plant Growth Promotion by Volatiles of Root‐Associated *Microbacterium* spp.” Applied and Environmental Microbiology 84, no. 22: e01865‐01818. 30194105 10.1128/AEM.01865-18 PMC6210106

[mbo370085-bib-0024] Cox, M. S. , P. D. Gerard , M. C. Wardlaw , and M. J. Abshire . 2003. “Variability of Selected Soil Properties and Their Relationships With Soybean Yield.” Soil Science Society of America Journal 67, no. 4: 1296–1302.

[mbo370085-bib-0025] Cui, H. , G. Wang , Y. Yang , Y. Yang , R. Chang , and F. Ran . 2016. “Soil Microbial Community Composition and Its Driving Factors in Alpine Grasslands Along a Mountain Elevational Gradient.” Journal of Mountain Science 13: 1013–1023.

[mbo370085-bib-0026] Donald, L. , A. Pipite , R. Subramani , J. Owen , R. A. Keyzers , and T. Taufa . 2022. “ *Streptomyces*: Still the Biggest Producer of New Natural Secondary Metabolites, a Current Perspective.” Microbiology Research 13, no. 3: 418–465.

[mbo370085-bib-0027] Enagbonma, B. J. , C. F. Ajilogba , and O. O. Babalola . 2020. “Metagenomic Profiling of Bacterial Diversity and Community Structure in Termite Mounds and Surrounding Soils.” Archives of Microbiology 202, no. 10: 2697–2709. 32725600 10.1007/s00203-020-01994-w

[mbo370085-bib-0028] Enagbonma, B. J. , and O. O. Babalola . 2019. “Potentials of Termite Mound Soil Bacteria in Ecosystem Engineering for Sustainable Agriculture.” Annals of Microbiology 69: 211–219.

[mbo370085-bib-0029] Enagbonma, B. J. , and O. O. Babalola . 2020. “Unveiling Plant‐Beneficial Function as Seen in Bacteria Genes From Termite Mound Soil.” Journal of Soil Science and Plant Nutrition 20, no. 2: 421–430.

[mbo370085-bib-0030] Enagbonma, B. J. , and O. O. Babalola . 2022. “Metagenomics Shows That Termite Activities Influence the Diversity and Composition of Soil Invertebrates in Termite Mound Soils.” Applied and Environmental Soil Science 2022, no. 1: 1–9.

[mbo370085-bib-0031] Enagbonma, B. J. , and O. O. Babalola . 2023. “Metagenomics Reveals the Microbiome Multifunctionalities of Environmental Importance From Termite Mound Soils.” Bioinformatics and Biology Insights 17: 11779322231184025. 37424707 10.1177/11779322231184025 PMC10328015

[mbo370085-bib-0032] Enagbonma, B. J. , A. E. Fadiji , and O. O. Babalola . 2024. “Anthropogenic Fertilization Influences a Shift in Barley Rhizosphere Microbial Communities.” PeerJ 12: e17303. 39006020 10.7717/peerj.17303 PMC11246026

[mbo370085-bib-0033] Enagbonma, B. J. , E. E. Imade , O. O. Babalola , D. M. Modise , E. C. Rigobelo , and C. Cruz . 2025. “Exploring Plant–Microbe Interactions in Extreme Environments: Lessons From Arid and Desert Ecosystems.” In Microbial Allies, edited by O. O. Babalola and A. S. Ayangbenro , 49–65. Springer. 10.1007/978-3-031-90530-8_3.

[mbo370085-bib-0034] Fan, Y. , Z. Zhou , F. Liu , et al. 2024. “The Vertical Partitioning Between Denitrification and Dissimilatory Nitrate Reduction to Ammonium of Coastal Mangrove Sediment Microbiomes.” Water Research 262: 122113. 39032335 10.1016/j.watres.2024.122113

[mbo370085-bib-0035] Farouq, A. A. , H. Y. Ismail , A. B. Rabah , A. B. Muhammad , U. B. Ibrahim , and A. Y. Fardami . 2022. “Cowpea Induced Physicochemical and Biological Rhizosphere Changes in Hydrocarbon Contaminated Soil.” Plant and Soil 477, no. 1: 759–777.

[mbo370085-bib-0036] Forbes, M. S. , K. Broos , J. A. Baldock , A. L. Gregg , and S. A. Wakelin . 2009. “Environmental and Edaphic Drivers of Bacterial Communities Involved in Soil N‐Cycling.” Soil Research 47, no. 4: 380–388.

[mbo370085-bib-0037] Franche, C. , K. Lindström , and C. Elmerich . 2009. “Nitrogen‐Fixing Bacteria Associated With Leguminous and Non‐Leguminous Plants.” Plant and Soil 321: 35–59.

[mbo370085-bib-0038] Fu, B. , L. Chen , H. Huang , P. Qu , and Z. Wei . 2021. “Impacts of Crop Residues on Soil Health: A Review.” Environmental Pollutants and Bioavailability 33, no. 1: 164–173.

[mbo370085-bib-0039] Gemayel, K. , A. Lomsadze , and M. Borodovsky . 2022. MetaGeneMark‐2: Improved Gene Prediction in Metagenomes. BioRxiv:2022.2007. 2025.500264. 10.1101/2022.07.25.500264.

[mbo370085-bib-0040] Geng, S. , J. Tan , L. Li , Y. Miao , and Y. Wang . 2023. “Legumes Can Increase the Yield of Subsequent Wheat With or Without Grain Harvesting Compared to Gramineae Crops: A Meta‐Analysis.” European Journal of Agronomy 142: 126643.

[mbo370085-bib-0041] Ghosh, P. K. , K. K. Hazra , M. S. Venkatesh , et al. 2020. “Grain Legume Inclusion in Cereal–Cereal Rotation Increased Base Crop Productivity in the Long Run.” Experimental Agriculture 56, no. 1: 142–158.

[mbo370085-bib-0042] Greenlon, A. , P. L. Chang , Z. M. Damtew , et al. 2019. “Global‐Level Population Genomics Reveals Differential Effects of Geography and Phylogeny on Horizontal Gene Transfer in Soil Bacteria.” Proceedings of the National Academy of Sciences 116, no. 30: 15200–15209. 10.1073/pnas.1900056116 PMC6660780 31285337

[mbo370085-bib-0043] Grzyb, A. , A. Wolna‐Maruwka , and A. Niewiadomska . 2020. “Environmental Factors Affecting the Mineralization of Crop Residues.” Agronomy 10: 1951.

[mbo370085-bib-0044] Hale, V. L. , P. Jeraldo , J. Chen , et al. 2018. “Distinct Microbes, Metabolites, and Ecologies Define the Microbiome in Deficient and Proficient Mismatch Repair Colorectal Cancers.” Genome Medicine 10, no. 1: 78. 30376889 10.1186/s13073-018-0586-6 PMC6208080

[mbo370085-bib-0045] Hamza, A. , and E. A. Akinrinde . 2016. “Response of Sorghum (*Sorghum bicolor* L.) to Residual Phosphate in Soybean–Sorghum and Maize–Sorghum Crop Rotation Schemes on Two Contrasting Nigerian Alfisols.” International Journal of Agronomy 2016, no. 1: 6945024.

[mbo370085-bib-0046] Hassen, A. , D. G. Talore , E. H. Tesfamariam , M. A. Friend , and T. D. E. Mpanza . 2017. “Potential Use of Forage‐Legume Intercropping Technologies to Adapt to Climate‐Change Impacts on Mixed Crop‐Livestock Systems in Africa: A Review.” Regional Environmental Change 17: 1713–1724.

[mbo370085-bib-0047] Hodge, A. , and A. H. Fitter . 2010. “Substantial Nitrogen Acquisition by Arbuscular Mycorrhizal Fungi From Organic Material Has Implications for N Cycling.” Proceedings of the National Academy of Sciences 107, no. 31: 13754–13759. 10.1073/pnas.1005874107 PMC2922220 20631302

[mbo370085-bib-0048] Hou, Z. , W. Dong , H. Wang , et al. 2023. “Response of Nitrite Accumulation to Elevated C/NO_3_‐ratio During Partial Denitrification Process: Insights of Extracellular Polymeric Substance, Microbial Community and Metabolic Function.” Microbial Community and Metabolic Function 384: 129269. 10.1016/j.biortech.2023.129269. 37290706

[mbo370085-bib-0049] Huson, D. H. , A. F. Auch , J. Qi , and S. C. Schuster . 2007. “MEGAN Analysis of Metagenomic Data.” Genome Research 17, no. 3: 377–386. 17255551 10.1101/gr.5969107 PMC1800929

[mbo370085-bib-0050] Imade, E. E. , S. E. Omonigho , O. O. Babalola , B. J. Enagbonma , O. N. Igiehon , and A. G. Ogofure . 2024. “Dataset of 16S Ribosomal DNA Sequence‐Based Identification of Bacteriocinogenic Lactic Acid Bacteria Isolated From Fermented Food Samples.” Data in Brief 52: 110021. 10.1016/j.dib.2023.110021. 38287954 PMC10823100

[mbo370085-bib-0051] Jalloh, A. A. , F. M. Khamis , A. A. Yusuf , S. Subramanian , and D. M. Mutyambai . 2024. “Long‐Term Push–Pull Cropping System Shifts Soil and Maize‐Root Microbiome Diversity Paving Way to Resilient Farming System.” BMC Microbiology 24, no. 1: 92. 38500045 10.1186/s12866-024-03238-z PMC10946131

[mbo370085-bib-0052] Jiao, S. , W. Chen , J. Wang , N. Du , Q. Li , and G. Wei . 2018. “Soil Microbiomes With Distinct Assemblies Through Vertical Soil Profiles Drive the Cycling of Multiple Nutrients in Reforested Ecosystems.” Microbiome 6: 146. 30131068 10.1186/s40168-018-0526-0 PMC6104017

[mbo370085-bib-0053] Karlsson, F. H. , F. Fåk , I. Nookaew , et al. 2012. “ *Symptomatic atherosclerosis* Is Associated With an Altered Gut Metagenome.” Nature Communications 3, no. 1: 1245. 10.1038/ncomms2266 PMC3538954 23212374

[mbo370085-bib-0054] Kelly, C. , M. L. Haddix , P. F. Byrne , et al. 2022. “Divergent Belowground Carbon Allocation Patterns of Winter Wheat Shape Rhizosphere Microbial Communities and Nitrogen Cycling Activities.” Soil Biology and Biochemistry 165: 108518.

[mbo370085-bib-0055] Khalifa, M. , and E. A. B. Eltahir . 2023. “Assessment of Global Sorghum Production, Tolerance, and Climate Risk.” Frontiers in Sustainable Food Systems 7: 1184373.

[mbo370085-bib-0056] Khoshru, B. , A. Fallah Nosratabad , V. A. J. Mahjenabadi , et al. 2025. “Multidimensional Role of *Pseudomonas*: From Biofertilizers to Bioremediation and Soil Ecology to Sustainable Agriculture.” Journal of Plant Nutrition 48, no. 6: 1016–1042.

[mbo370085-bib-0057] Kombat, R. K. , K. Gyasi Santo , K. Atakora , and A. A. Khalid . 2024. “Cowpea (*Vigna unguiculata* (L.)‐Maize (*Zea mays* L.) Intercropping and Fertilizer Application Affected Maize Yield and Yield Components in Guinea Savannah Agro‐Ecological Zone of Ghana.” Archives of Agronomy and Soil Science 70, no. 1: 1–19.

[mbo370085-bib-0058] Langmead, B. , and S. L. Salzberg . 2012. “Fast Gapped‐Read Alignment With Bowtie2.” Nature Methods 9, no. 4: 357–359. 22388286 10.1038/nmeth.1923 PMC3322381

[mbo370085-bib-0059] Lei, Z. , K. Zhang , C. Li , et al. 2019. “Ruminal Metagenomic Analyses of Goat Data Reveals Potential Functional Microbiota by Supplementation With Essential Oil–Cobalt Complexes.” BMC Microbiology 19: 30. 30717674 10.1186/s12866-019-1400-3 PMC6362596

[mbo370085-bib-0060] Li, D. , R. Luo , C.‐M. Liu , et al. 2016. “MEGAHIT v1. 0: A Fast and Scalable Metagenome Assembler Driven by Advanced Methodologies and Community Practices.” Methods 102: 3–11. 27012178 10.1016/j.ymeth.2016.02.020

[mbo370085-bib-0061] Li, S. , F. Xiao , D. Yang , et al. 2021. “Nitrate Transport and Distribution in Soybean Plants With Dual‐Root Systems.” Frontiers in Plant Science 12: 661054. 34093618 10.3389/fpls.2021.661054 PMC8174562

[mbo370085-bib-0062] Liu, C. , J. Wang , Y. Wang , et al. 2025. “Crop Rotation and Fertilization Shape the Microbiomes of Maize Rhizosphere Soil With Distinct Mechanisms.” Plant and Soil 507, no. 1: 89–108.

[mbo370085-bib-0063] Liu, L. , J. D. Knight , R. L. Lemke , and R. E. Farrell . 2024. “Quantifying the Contribution of Above‐ and Below‐Ground Residues of Chickpea, Faba Bean, Lentil, Field Pea and Wheat to the Nitrogen Nutrition of a Subsequent Wheat Crop.” Field Crops Research 313: 109412.

[mbo370085-bib-0064] Liu, W. , B. Schmidt , Y. Liu , G. Voss , and W. Mueller‐Wittig . 2021. “Mapping of BLASTP Algorithm onto GPU Clusters.” In China (ed) 2011 IEEE 17th International Conference on Parallel and Distributed Systems, Tainan, Taiwan, 236–243. IEEE. 10.1109/ICPADS.2011.79.

[mbo370085-bib-0065] Liu, X.‐Q. , M.‐M. Xie , A. Hashem , E. F. Abd‐Allah , and Q.‐S. Wu . 2023. “Arbuscular Mycorrhizal Fungi and Rhizobia Synergistically Promote Root Colonization, Plant Growth, and Nitrogen Acquisition.” Plant Growth Regulation 100, no. 3: 691–701.

[mbo370085-bib-0066] Marschner, P. , R. G. Joergensen , H. P. Piepho , and A. Buerkert . 2004. “Legume Rotation Effects on Early Growth and Rhizosphere Microbiology of Sorghum in West African Soils.” Plant and Soil 264, no. 1: 325–334.

[mbo370085-bib-0067] Matthews, L. , J. A. Strauss , T. Reinsch , et al. 2025. “Legumes and Livestock in No‐Till Crop Rotations: Effects on Nitrous Oxide Emissions, Carbon Sequestration, Yield, and Wheat Protein Content.” Agricultural Systems 224: 104218.

[mbo370085-bib-0068] Moore, B. D. , R. L. Andrew , C. Külheim , and W. J. Foley . 2014. “Explaining Intraspecific Diversity in Plant Secondary Metabolites in an Ecological Context.” New Phytologist 201, no. 3: 733–750. 24117919 10.1111/nph.12526

[mbo370085-bib-0069] Moreno, G. , A. Hernández‐Esteban , V. Rolo , and J. M. Igual . 2021. “The Enduring Effects of Sowing Legume‐Rich Mixtures on the Soil Microbial Community and Soil Carbon in Semi‐Arid Wood Pastures.” Plant and Soil 465, no. 1: 563–582.

[mbo370085-bib-0070] Mupangwa, W. , I. Nyagumbo , F. Liben , L. Chipindu , P. Craufurd , and S. Mkuhlani . 2021. “Maize Yields From Rotation and Intercropping Systems With Different Legumes Under Conservation Agriculture in Contrasting Agro‐Ecologies.” Agriculture, Ecosystems & Environment 306: 107170.

[mbo370085-bib-0071] N'Dayegamiye, A. , J. K. Whalen , G. Tremblay , et al. 2015. “The Benefits of Legume Crops on Corn and Wheat Yield, Nitrogen Nutrition, and Soil Properties Improvement.” Agronomy Journal 107, no. 5: 1653–1665.

[mbo370085-bib-0072] Natta, G. , S. Voyron , E. Lumini , et al. 2025. “Gut Microbiota Variability in Dung Beetles: Prokaryotes Vary According to the Phylogeny of the Host Species While Fungi Vary According to the Diet.” Frontiers in Insect Science 5: 2025. 10.3389/finsc.2025.1639013. PMC12405213 40908959

[mbo370085-bib-0073] Nguyen, T. T. , T. R. Cavagnaro , H. T. Thanh Ngo , and P. Marschner . 2016. “Soil Respiration, Microbial Biomass and Nutrient Availability in Soil Amended With High and Low C/N Residue—Influence of Interval Between Residue Additions.” Soil Biology and Biochemistry 95: 189–197.

[mbo370085-bib-0074] Ogundijo, D. , M. Adetunji , J. Azeez , T. Arowolo , N. Olla , and A. Adekunle . 2015. “Influence of Organic and Inorganic Fertilizers on Soil Chemical Properties and Nutrient Changes in an Alfisol of South Western Nigeria.” International Journal of Plant & Soil Science 7, no. 6: 329–337.

[mbo370085-bib-0075] Ojiem, J. O. , A. C. Franke , B. Vanlauwe , N. De Ridder , and K. E. Giller . 2014. “Benefits of Legume–Maize Rotations: Assessing the Impact of Diversity on the Productivity of Smallholders in Western Kenya.” Field Crops Research 168: 75–85.

[mbo370085-bib-0076] Oksanen, J. , R. Kindt , P. Legendre , et al. 2016. “vegan: An R Package for Community Ecologists.” R Package Version 14, no. 41: 37.

[mbo370085-bib-0077] Pambuka, G. T. , T. R. Kinge , S. Ghosh , E. D. Cason , M. M. Nyaga , and M. Gryzenhout . 2021. “Baseline Data of the Fungal Phytobiome of Three Sorghum (*Sorghum bicolor*) Cultivars in South Africa Using Targeted Environmental Sequencing.” Journal of Fungi 7, no. 11: 978. 34829265 10.3390/jof7110978 PMC8622221

[mbo370085-bib-0078] Pappa, V. A. , R. M. Rees , R. L. Walker , J. A. Baddeley , and C. A. Watson . 2012. “Legumes Intercropped With Spring Barley Contribute to Increased Biomass Production and Carry‐Over Effects.” Journal of Agricultural Science 150, no. 5: 584–594.

[mbo370085-bib-0079] Petchey, O. L. , and K. J. Gaston . 2006. “Functional Diversity: Back to Basics and Looking Forward.” Ecology Letters 9, no. 6: 741–758. 10.1111/j.1461-0248.2006.00924.x. 16706917

[mbo370085-bib-0080] Qi, W. , Q. Wang , E. Mak‐Mensah , et al. 2024. “Effects of Soil Physicochemical Properties on Maize, Wheat, and Soybean Yields in Maize–Wheat and Maize–Soybean Intercropping Systems in China: A Meta‐Analysis.” Journal of Soil Science and Plant Nutrition 24, no. 1: 21–29.

[mbo370085-bib-0081] Rao, C. R. 1964. “The Use and Interpretation of Principal Component Analysis in Applied Research.” Sankhyā: The Indian Journal of Statistics, Series A 26: 329–358.

[mbo370085-bib-0082] Rao, D. , F. Meng , X. Yan , et al. 2021. “Changes in Soil Microbial Activity, Bacterial Community Composition and Function in a Long‐Term Continuous Soybean Cropping System After Corn Insertion and Fertilization.” Frontiers in Microbiology 12: 638326. 33897643 10.3389/fmicb.2021.638326 PMC8059791

[mbo370085-bib-0083] Sebetha, E. , A. Modi , and L. Owoeye . 2015. “Cowpea Crude Protein as Affected by Cropping System, Site and Nitrogen Fertilization.” Journal of Agricultural Science 7, no. 1: 224.

[mbo370085-bib-0084] Shah, K. K. , B. Modi , H. P. Pandey , et al. 2021. “Diversified Crop Rotation: An Approach for Sustainable Agriculture Production.” Advances in Agriculture 2021, no. 1: 8924087.

[mbo370085-bib-0085] Shu, D. , S. Banerjee , X. Mao , et al. 2024. “Conversion of Monocropping to Intercropping Promotes Rhizosphere Microbiome Functionality and Soil Nitrogen Cycling.” Science of the Total Environment 949: 174953. 39069174 10.1016/j.scitotenv.2024.174953

[mbo370085-bib-0086] Shuaibu, Y. , A. Garba , and N. Voncir . 2015. “Influence of Legume Residue and Nitrogen Fertilizer on the Growth and Yield of Sorghum (*Sorghum bicolor* (L.) Moench) in Bauchi State, Nigeria.” African Journal of Food, Agriculture, Nutrition and Development 15, no. 3: 10060–10076.

[mbo370085-bib-0087] Siczek, A. , A. Gryta , K. Oszust , and M. Frąc . 2024. “Faba Bean in Crop Rotation Shapes Bacterial and Fungal Communities and Nutrient Contents Under Conventional Tillage of Triticale.” Applied Soil Ecology 202: 105597.

[mbo370085-bib-0088] Suresh, G. , R. Raghavan , and A. S. Narayanan . 2025. “Microbiome Analysis of Metagenome Using RStudio.” In Plant Microbiome Engineering. Methods and Protocols in Food Science, edited by D. Dharumadurai and A. S. Narayanan , 323–340. Humana. 10.1007/978-1-0716-4180-4_39.

[mbo370085-bib-0089] Tonapi, V. A. , H. S. Talwar , A. K. Are , B. V. Bhat , C. R. Reddy , and T. J. Dalton . 2020. Sorghum in the 21st Century: Food, Fodder, Feed, Fuel for a Rapidly Changing World. Springer.

[mbo370085-bib-0090] Torma, S. , J. Vilček , T. Lošák , S. Kužel , and A. Martensson . 2018. “Residual Plant Nutrients in Crop Residues—An Important Resource.” Acta Agriculturae Scandinavica, Section B—Soil & Plant Science 68, no. 4: 358–366.

[mbo370085-bib-0091] Tu, Q. , L. Lin , L. Cheng , Y. Deng , and Z. He . 2019. “NCycDB: A Curated Integrative Database for Fast and Accurate Metagenomic Profiling of Nitrogen Cycling Genes.” Bioinformatics 35, no. 6: 1040–1048. 30165481 10.1093/bioinformatics/bty741

[mbo370085-bib-0092] Tzemi, D. , J. Rämö , T. Palosuo , P. Peltonen‐Sainio , H. Wejberg , and H. Lehtonen . 2024. “The Introduction of Legume‐Based Crop Rotations: An Impact Assessment on Cereal Cropping Farms in Finland.” International Journal of Agricultural Sustainability 22, no. 1: 2335085.

[mbo370085-bib-0093] Ugochukwu, E. J. , and P. U. Okorie . 2020. “Isolation and Characterization of Streptomycetes With Potential to Decompose Organic Compounds During Bioremediation of Arable Soil.” Sustinere: Journal of Environment and Sustainability 4, no. 1: 16–23.

[mbo370085-bib-0094] Uzoh, I. M. , C. A. Igwe , C. B. Okebalama , and O. O. Babalola . 2019. “Legume–Maize Rotation Effect on Maize Productivity and Soil Fertility Parameters Under Selected Agronomic Practices in a Sandy Loam Soil.” Scientific Reports 9, no. 1: 8539. 31189881 10.1038/s41598-019-43679-5 PMC6561941

[mbo370085-bib-0095] Villar, E. , G. K. Farrant , M. Follows , et al. 2015. “Environmental Characteristics of Agulhas Rings Affect Interocean Plankton Transport.” Science 348, no. 6237: 1261447. 25999514 10.1126/science.1261447

[mbo370085-bib-0096] Wang, S. , S. Guo , L. Zhai , et al. 2022. “Comprehensive Effects of Integrated Management on Reducing Nitrogen and Phosphorus Loss Under Legume–Rice Rotations.” Journal of Cleaner Production 361: 132031.

[mbo370085-bib-0097] Wang, X.‐X. , X. Wang , Y. Sun , et al. 2018. “Arbuscular Mycorrhizal Fungi Negatively Affect Nitrogen Acquisition and Grain Yield of Maize in a N Deficient Soil.” Frontiers in Microbiology 9: 418. 29568292 10.3389/fmicb.2018.00418 PMC5852317

[mbo370085-bib-0098] Wang, P. , W. Xie , L. Ding , et al. 2023. “Effects of Maize–Crop Rotation on Soil Physicochemical Properties, Enzyme Activities, Microbial Biomass and Microbial Community Structure in Southwest China.” Microorganisms 11, no. 11: 2621. 38004632 10.3390/microorganisms11112621 PMC10672910

[mbo370085-bib-0099] Wang, Y. , L. Zhang , F. Meng , et al. 2023. “Responses of Soil Microbial Communities in Soybean–Maize Rotation to Different Fertilization Treatments.” Agronomy 13, no. 6: 1590.

[mbo370085-bib-0100] Wendeborn, S. 2020. “The Chemistry, Biology, and Modulation of Ammonium Nitrification in Soil.” Angewandte Chemie International Edition 59, no. 6: 2182–2202. 31116902 10.1002/anie.201903014

[mbo370085-bib-0101] Yan, L. , Y. Kuang , X. Xie , et al. 2024. “Insights into Nitrogen Biogeochemical Cycling in Mangrove Wetland From Genome‐Resolved Metagenomic Sequencing.” Journal of Hydrology 640: 131741.

[mbo370085-bib-0102] Yu, H. , F. Wang , M. Shao , et al. 2021. “Effects of Rotations With Legume on Soil Functional Microbial Communities Involved in Phosphorus Transformation.” Frontiers in Microbiology 12: 661100. 34659135 10.3389/fmicb.2021.661100 PMC8519609

[mbo370085-bib-0103] Yu, K. , and T. Zhang . 2013. “Construction of Customized Sub‐Databases From NCBI‐nr Database for Rapid Annotation of Huge Metagenomic Datasets Using a Combined BLAST and MEGAN Approach.” PLoS ONE 8, no. 4: e59831. 23573212 10.1371/journal.pone.0059831 PMC3613424

[mbo370085-bib-0104] Yusuf, A. A. , R. C. Abaidoo , E. N. O. Iwuafor , and O. O. Olufajo . 2008. “Genotype Effects of Cowpea and Soybean on Nodulation, N_2_‐fixation and N Balance in the Northern Guinea Savanna of Nigeria.” Journal of Agronomy 7, no. 3: 258–264. 10.3923/ja.2008.258.264.

[mbo370085-bib-0105] Zang, H. , X. Qian , Y. Wen , et al. 2018. “Contrasting Carbon and Nitrogen Rhizodeposition Patterns of Soya Bean (*Glycine max* L.) and Oat (*Avena nuda* L.).” European Journal of Soil Science 69, no. 4: 625–633.

[mbo370085-bib-0106] Zeller, G. , J. Tap , A. Y. Voigt , et al. 2014. “Potential of Fecal Microbiota for Early‐Stage Detection of Colorectal Cancer.” Molecular Systems Biology 10, no. 11: 766. 25432777 10.15252/msb.20145645 PMC4299606

[mbo370085-bib-0107] Zhao, Y. , S. Guo , X. Zhu , et al. 2024. “How Maize–Legume Intercropping and Rotation Contribute to Food Security and Environmental Sustainability.” Journal of Cleaner Production 434: 140150.

[mbo370085-bib-0108] Zhong, Y. , J. Tian , X. Li , and H. Liao . 2023. “Cooperative Interactions Between Nitrogen Fixation and Phosphorus Nutrition in Legumes.” New Phytologist 237, no. 3: 734–745. 36324147 10.1111/nph.18593

[mbo370085-bib-0109] Zhou, T. , P. Wu , C. Yue , J. Huang , Z. Zhang , and Y. Hua . 2022. “Transcriptomic Dissection of Allotetraploid Rapeseed (*Brassica napus* L.) in Responses to Nitrate and Ammonium Regimes and Functional Analysis of BnaA2. Gln1; 4 in Arabidopsis.” Plant and Cell Physiology 63, no. 6: 755–769. 35325216 10.1093/pcp/pcac037

