# Supplementary material for: Effects of Legume‒Cereal Rotation on Sorghum Rhizosphere Microbial Community Structure and Nitrogen‐Cycling Functions
Source: Microbiologyopen. 2025 Oct 21;14(5):e70085. doi: 10.1002/mbo3.70085 (PMC12540930; doi:10.1002/mbo3.70085)
Supplement: Supplementary file 1 — MicrobiologyOpen supplimentary_revised. [file MBO3-14-e70085-s001.docx]

**Sorghum plant cultivation in soils preceded by legumes promotes rhizosphere microbiome functionality and soil nitrogen cycling**

^1^Ben Jesuorsemwen Enagbonma (<https://orcid.org/0000-0002-4699-5061>), ^2^David Mxolisi Modise (<https://orcid.org/0000-0001-5381-9472>), and ^1,3^Olubukola Oluranti Babalola* (<https://orcid.org/0000-0003-4344-1909>)

Table S1: Quality control summary of the sequences across all different cropping system

| **Group** | **G1** | **G2** | **G3** | **G4** | **G5** | **G6** | **G7** | **G8** |
| --- | --- | --- | --- | --- | --- | --- | --- | --- |
| Raw_Base (G) | 10.47333 | 9.29 | 9.493333 | 10.25333 | 10.88333 | 10.43333 | 8.626667 | 12.03 |
| Clean_Base (G) | 10.44333 | 9.27 | 9.46 | 10.22333 | 10.85333 | 10.4 | 8.6 | 12 |
| Clean_Q20 (%) | 97.89667 | 97.98667 | 97.52333 | 97.63333 | 97.57333 | 97.33 | 97.41667 | 97.43 |
| Clean_Q30 (%) | 94.47667 | 94.72667 | 93.71333 | 94.05333 | 93.93 | 93.24333 | 93.35333 | 93.50333 |
| Clean_GC (%) | 67.36 | 67.15 | 67.35 | 67.04 | 67.45 | 67.29667 | 66.55667 | 67.44667 |
| Effective (%) | 99.69333 | 99.74333 | 99.63333 | 99.73667 | 99.73667 | 99.63 | 99.66667 | 99.77333 |

**
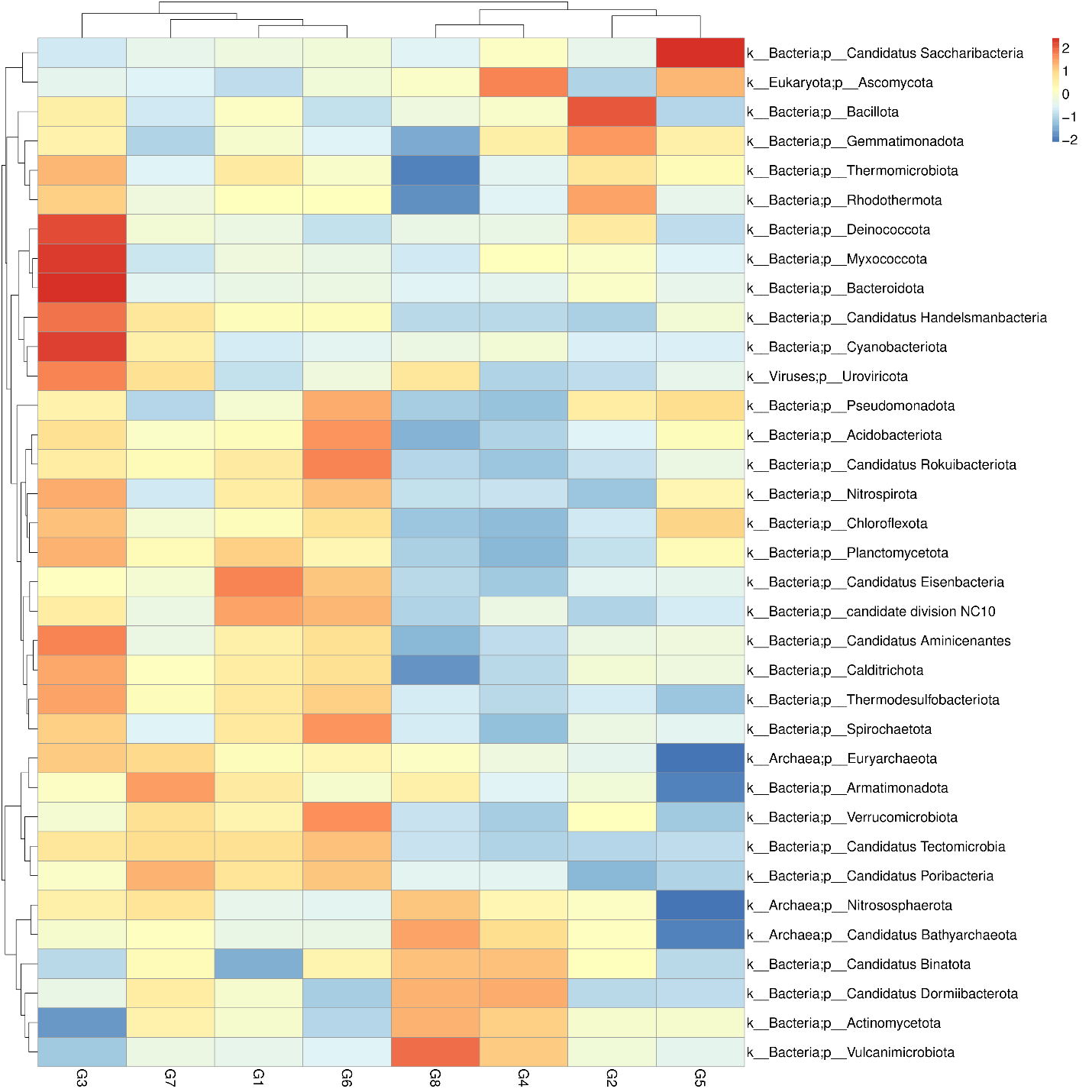
**

Fig. S1: Heatmap showcases the relative abundance of various microbial taxa across the cropping system. The color bar on the right indicates relative abundances where red represents high relative abundance, blue signifies low relative abundance, yellow and light shades show intermediate levels. K stands for kingdom while p stands for phylum


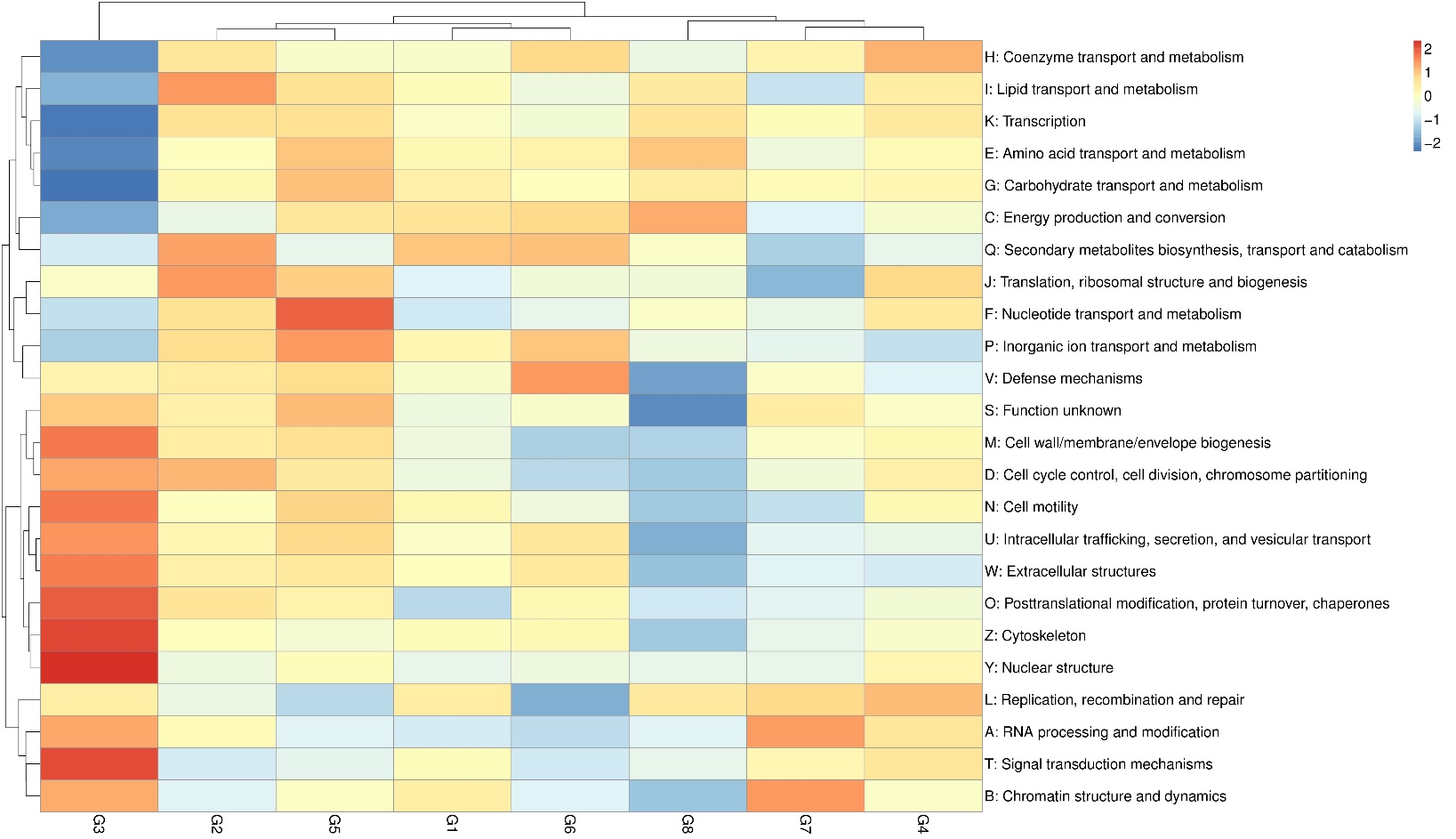


Fig S2: Functional groups depicted by eggNOG databases across all different cropping system


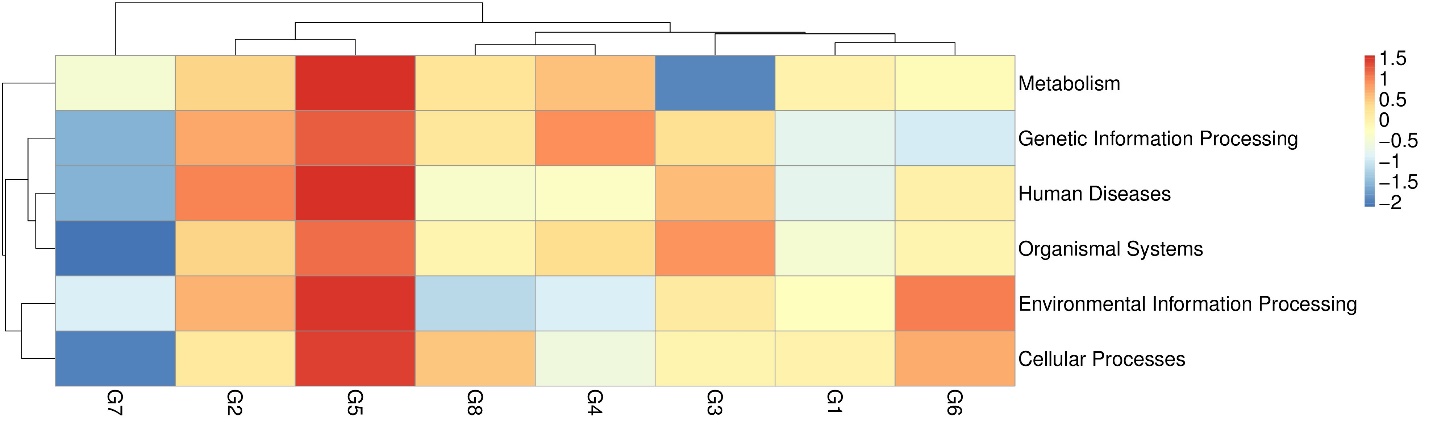


Fig S3: Functional groups depicted by KEGG databases across all different cropping system


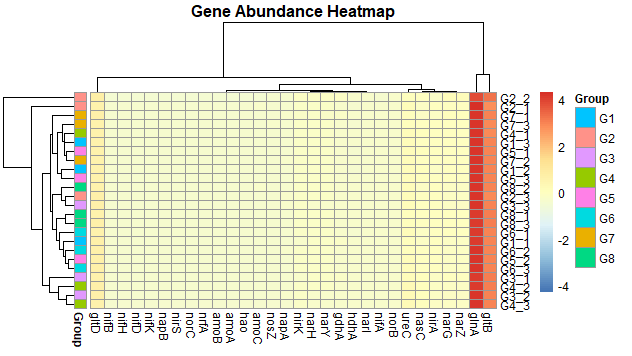


Fig S4: The distribution of functional genes involved in nitrogen cycling across the cropping system. The color bar on the right indicates relative abundances where red represents high relative abundance, blue signifies low relative abundance

Table S2: RDA analysis

| **Soil variable** | **RDA1** | **RDA2** | **r²** | **Pr(>r)** | **Significance** |
| --- | --- | --- | --- | --- | --- |
| pH | -0.91339 | -0.40708 | 0.5602 | 0.137 |  |
| P | -0.54493 | -0.83848 | 0.2181 | 0.459 |  |
| K...4 | 0.91357 | -0.40667 | 0.4140 | 0.271 |  |
| *S | -0.98449 | -0.17547 | 0.6298 | 0.096 | . |
| K...6 | 0.99976 | -0.02195 | 0.5942 | 0.134 |  |
| Ca | 0.89027 | 0.45544 | 0.4918 | 0.223 |  |
| Mg | -0.99669 | -0.08135 | 0.5731 | 0.131 |  |
| Na | -0.75244 | -0.65866 | 0.3725 | 0.312 |  |
| N-NO_3_ | -0.75366 | 0.65726 | 0.8603 | 0.008 | ** |
| N-NH_4_ | -0.94867 | 0.31628 | 0.0711 | 0.858 |  |
| Sand | 0.57492 | -0.81821 | 0.6933 | 0.046 | * |
| Silt | 0.87804 | 0.47859 | 0.9373 | 0.002 | ** |
| Clay | -0.96106 | -0.27633 | 0.9397 | 0.004 | ** |
| OC | 0.93094 | 0.36518 | 0.1199 | 0.778 |  |
